# Enhanced DEWMA-type control chart for process mean monitoring utilizing auxiliary information

**DOI:** 10.1038/s41598-025-27540-6

**Published:** 2025-12-15

**Authors:** Saadia Masood, Khawar Ibrar, Zabihullah Movaheedi, Hafsa Jabeen

**Affiliations:** 1 Department of Statistics, PMAS-University of Arid Agriculture Rawalpindi, Rawalpindi, Pakistan; 2 Department of Mathematics, Faculty of Science, Herat University, Herat, Afghanistan 3001

**Keywords:** Applied mathematics, Statistics, Mathematics and computing

## Abstract

Statistical Process Control (SPC) improves product quality by monitoring process performance, with control charts being the primary tool to detect and manage variability. The effectiveness of a control chart can be enhanced by incorporating additional pertinent information regarding the study variable. This study revisits the DEWMA chart, which is designed to monitor variations in the process mean under the assumption that the underlying process follows a normal distribution. We propose a Regression-based DEWMA (M_R_DEWMA) control chart that utilizes an auxiliary variable through a regression estimation method to determine the process mean. The control limits for the proposed chart are established based on both time-varying and asymptotic conditions. The features of run length (RL), including Average Run Length (ARL), Standard Deviation of Run Length (SDRL), and Median Run Length (MRL), are evaluated using Monte Carlo simulations. A comparative analysis reveals that the proposed M_R_DEWMA chart outperforms the traditional DEWMA chart in detecting small to moderate shifts in the process mean. The efficacy of the proposed approach is demonstrated using both Monte Carlo simulated data and a real-life industrial case study.

**Supplementary Information:**

The online version contains supplementary material available at 10.1038/s41598-025-27540-6.

## Introduction

Statistical Process Control (SPC) is a quantitative methodology vital for ensuring quality and consistency in both manufacturing and non-manufacturing environments. It encompasses seven fundamental tools: the Pareto diagram, cause-and-effect diagram, check sheets, process flow diagram, scatter diagram, histogram, and control chart. Among these, control charts are particularly effective in monitoring process variability and assessing process stability.

Control charts help determine whether a process is operating under a state of statistical control or exhibiting instability. Variations in a process are broadly categorized into two types: random (common) causes and non-random (assignable) causes. Random causes represent natural, inherent fluctuations that arise from many minor, uncontrollable sources and are expected in a stable process. In contrast, non-random causes stem from specific, identifiable factors that disrupt normal operations, indicating underlying issues requiring corrective action. Control charts are categorized into two primary types according to their design and structure: memory-less and memory-type control charts. The memory-less chart, identified as a Shewhart-type control chart, is ineffective in monitoring small or moderate shifts in the process. Another limitation of memory-less charts is their focus on the most recent sample observation, which leads to the exclusion of the previous sequence of values, thus relying solely on current information. To overcome this problem, Page^1^, Page^2^ and Roberts^3^ introduced the CUSUM and EWMA control chart.

The CUSUM chart can be classified into two types; the first type features a known direction of the shift, while the second type has a direction that is either undefined or unspecified. The CUSUM chart is proficient in identifying small to moderate shifts in the process. However, a significant limitation of the CUSUM chart is the difficulty it presents during implementation. Additionally, its speed of detection is not optimal for larger shifts. Hawkins et al.^4^ provided thorough information on the structure of the CUSUM chart and its Average Run Length (ARL) performance across various parameter settings. Another variant of memory-based control charts is the Exponentially Weighted Moving Average (EWMA) chart, which was introduced by Roberts^3^. The EWMA chart incorporates both current and historical data by assigning exponentially decreasing weights through a smoothing parameter. It emphasizes recent observations while progressively reducing the impact of older data. The effectiveness of the EWMA chart is highly dependent on the appropriate selection of the smoothing parameter. Notably, the EWMA chart follows the structure of a Markov process. To obtain a reliable estimate of the Average Run Length (ARL), one can utilize the Markov property or calculate the average Run Length using Monte Carlo simulation; please see Chen et al.^5^ and Zi et al.^6^.

Auxiliary information has not yet been integrated into the DEWMA chart framework for monitoring location parameters. This represents a clear research gap: by failing to leverage relevant supplementary variables, existing DEWMA charts may not achieve their full potential in terms of sensitivity and accuracy. Consequently, this study is motivated by the need to develop an M_R_DEWMA chart that incorporates auxiliary information, thereby enhancing its ability to detect subtle shifts in the process mean. Addressing this gap will not only enhance the statistical efficiency of the control chart but also broaden its practical applicability in quality monitoring and process improvement. Different performance metrics, including the ARL, SDRL, and MRL, are utilized to assess the effectiveness of proposed and existing charts. Of these metrics, ARL is the most frequently used. It indicates the average count of observations required before the chart indicates an out-of-control (OOC) situation. The ARL for in-control (IC) process is represented as ARL_0_, whereas ARL_1_ signifies the ARL when the process is deemed OOC.

In survey sampling, auxiliary information is frequently utilized for the design and estimation of unknown population parameters. Various estimators—such as the classical ratio, product, and regression estimators—are based on this auxiliary information. These estimators rely on data from both the study variable and one or more correlated auxiliary variables, thereby offering improved efficiency over estimators that depend exclusively on the study variable. Several efficient ratio-type estimators for estimating the population mean have been proposed by Kadilar et al.^7^, Srivastava et al.^8^, and Cochran^9^, among others. Auxiliary information plays a critical role in quality control by improving the effectiveness of control charts. Control charts are statistical tools utilized to monitor and regulate processes over time, and the incorporation of auxiliary information provides decision-makers with deeper insights into the processes being evaluated. For instance, auxiliary data may indicate that an unexpected deviation on the control chart arises from a change in the quality of raw materials or an adjustment in machine settings. While control charts primarily focus on tracking process variability, auxiliary information helps identify factors that are not directly monitored by the chart but still have a significant impact on overall performance. This perspective is supported by the research findings of Tatum et al.^10^, Zobel et al.^11^, Sukthomya et al.^12^ and Kourti et al.^13^, among others. Riaz^14^ and Riaz^15^ introduced auxiliary-based control charts. The Shewhart-type control charts employed in the works of Riaz^14,15^ have proven effective in detecting substantial shifts in process parameters. Subsequently Riaz et al.^16^ developed variability chart which utilized a ratio-type estimator, establishing its worth over the regression-type estimator-based chart.

The M_X_EWMA control chart, developed by Abbas et al.^17^, is a new EWMA-type control chart that utilizes a regression estimator with a single auxiliary variable. Haq et al.^18^ proposed the new synthetic control chart marks a significant advancement in process monitoring by using additional information to enhance detection and outperform traditional methods. Saghir et al.^19^ highlight the benefits of an EWMA control chart that utilizes auxiliary information and repeated sampling, demonstrating its effectiveness in process monitoring and the significance of correlation coefficients in its design. Haq et al.^20^ introduces a new method that combines the conforming RL chart with the established auxiliary-information-based (AIB) double sampling chart, creating the AIB synthetic double sampling (DS) chart for monitoring process means. This integration facilitates a comprehensive analysis of Run Length (RL) profiles associated with the proposed control chart. The findings indicate that the optimal AIB synthetic DS chart surpasses the existing AIB Shewhart, optimal AIB synthetic, and AIB DS charts in detecting shifts in the process mean, marking a substantial advancement in process monitoring methodologies. Moreover, this chart effectively detects small to moderate shifts in the process location parameter.

Recent studies have significantly contributed to enhancing process mean monitoring through the development of improved control charts. Javed et al.^21^ proposed a novel variant of the EWMA chart, improving sensitivity for mean shift detection. Similarly, Ng et al.^22^ developed an auxiliary information-based hybrid exponentially weighted moving average (HEWMA) chart with variable sampling intervals, further optimizing the detection capabilities under adaptive sampling schemes. Saha et al.^23^ analyzed the performance of X̄ and EWMA charts using auxiliary information specifically for short production runs, emphasizing their robustness in limited sample scenarios. Sanaullah et al.^24^ proposed an extended-EWMA control chart incorporating auxiliary information, which demonstrated increased efficiency in detecting small shifts. Collectively, these contributions highlight the critical role of auxiliary information in enhancing the sensitivity and responsiveness of statistical process control techniques. Cui et al.^25^ proposes a non-parametric triple generally weighted moving average sign chart, offering an effective alternative for process monitoring when underlying distributional assumptions are not met.

The organization of the paper is as follows: Section "EWMA control chart" describes the structure and functioning of the traditional EWMA control chart, while Section "DEWMA control chart" introduces the DEWMA control chart along with its development and benefits. Section "The proposed MR DEWMA control chart" presents the proposed M_R_DEWMA control chart, which incorporates regression-based auxiliary information. Section "Performance evaluation" outlines the performance evaluation criteria, with emphasis on run-length properties. Section "Simulation study" details the simulation study conducted using Monte Carlo methods to compare the proposed and existing charts. Section "Results and discussion" provides a comprehensive discussion of results based on ARL, SDRL, and MRL metrics. Section "Conclusion" concludes the study by summarizing key findings and practical implications. Lastly, Section "Future recommendations" offers future recommendations to further improve and expand the proposed methodology.

### EWMA control chart

The geometric moving average chart was the original term for the exponentially weighted moving average (EWMA) chart, Robert^3^ was presented. The name was changed to indicate that the base of EWMA chart is exponential smoothing. Let Y represent a quality attribute of a process having a normal distribution, with mean ($\mu$) and variance ($\sigma^{2}$) and let $Y_{t}$ **, **$t = 1,2,3.....,$ denoted the series of independent, identically distributed (iid) process observations. The IC process is defined as $\mu = \mu_{0}$; otherwise $\mu = \mu_{1} \ne \mu_{0}$. We assume that the process variance is IC and variance remains constant. We are interested in detecting a shift in the process mean from its IC value $\mu_{0}$ to an OOC value $\mu_{1} = \mu_{0} + \delta \sigma$, for $\delta \ne 0$. The EWMA statistics is defined as:

1 $$Z_{t} = \upsilon Y_{t} + (1 - \upsilon )Z_{(t - 1)}$$

where $Z_{t}$ is EWMA statistic at time *t*, $Z_{t - 1}$ is the past information at time $t - 1$, $Y_{t}$ is the observed value at time *t*, $\upsilon \in (0,1]$ is smoothing parameter and $Z_{0}$ is the initial value, often set to the process mean $\mu$.

The smoothing parameter *h* governs the degree of weighting applied to the information; a smaller value of *h* implies diminished influence, whereas values approaching unity reflect increased weighting in the estimation process. If $\upsilon = 1$, all the weight is given to the current observation, and the chart reduces to a Shewhart $\overline{X} - Chart$. The IC mean and variance are defined as:

2 $$E(Z_{t} ) = \mu_{0} {\text{ and}}\;V(Z_{t} ) = \sigma_{Y}^{2} \left( {\frac{\upsilon }{(2 - \upsilon )}(1 - (1 - \upsilon )^{2t} )} \right)$$

If the mean and standard deviation are unknown, they can be estimated from the sample values. The EWMA statistic, $Z_{t}$, is then plotted against the sample number (or time) to develop the EWMA control chart. The center line and control limits of the EWMA control chart are specified as follows:

3 $$\left. \begin{aligned} LCL_{t} = \mu_{0} - L\sigma_{Y} \sqrt {\frac{\upsilon }{(2 - \upsilon )}(1 - (1 - \upsilon )^{2t} )} \hfill \\ CL = \mu_{0} \hfill \\ UCL_{t} = \mu_{0} + L\sigma_{Y} \sqrt {\frac{\upsilon }{(2 - \upsilon )}(1 - (1 - \upsilon )^{2t} )} \hfill \\ \end{aligned} \right\}$$

In Eq. (3), *L* represents the width of the control limits, which remain invariant with respect to sample size. As the time index increases, the associated term asymptotically approaches unity, indicating convergence of the control limits to their steady-state values. This behavior characterizes the long-run stability and performance consistency of the EWMA control chart throughout the monitoring process.

4 $$\left. \begin{aligned} LCL = \mu_{0} - L\sigma_{Y} \sqrt {\frac{\upsilon }{(2 - \upsilon )}} \hfill \\ CL = \mu_{0} \hfill \\ UCL = \mu_{0} + L\sigma_{Y} \sqrt {\frac{\upsilon }{(2 - \upsilon )}} \hfill \\ \end{aligned} \right\}$$

Equation (4) defines the asymptotic control limits, whereas the preceding equation specifies the time-varying limits. The time-varying limits incorporate the exact control limit width for early observations. Consequently, an EWMA control chart utilizing time-varying limits exhibits enhanced sensitivity to initial out-of-control (OOC) conditions.

### DEWMA control chart

The first control chart utilizing a double exponentially weighted moving average (DEWMA) was proposed and evaluated by Shamma et al.^26^. A double exponentially weighted moving average control chart (DEWMA) approach was later developed by Zhang et al.^27^ as an extension of the EWMA technique. Both studies arrived at similar conclusions: when the process mean shift is less than half of the process standard deviation, the DEWMA mean chart outperforms the EWMA mean chart. For larger mean shifts, the performance of EWMA and DEWMA charts is comparable. Building on these findings, several studies explored various aspects of DEWMA charts. Notably Alkahtani et al.^28^ assessed the robustness of both DEWMA and EWMA charts under non-normal process conditions. Alkahtani et al.^29^ developed a multivariate DEWMA control chart to detect shifts in the mean vector of a multivariate normal distribution of quality characteristics.

The DEWMA chart represents advancement over the traditional EWMA chart, as it introduces a second degree of exponential smoothing to better detect small and moderate changes in the process mean. This approach is particularly useful when the process requires enhanced stability against short-term variations. Let the sequence of observed data be $Y_{t}$ for $t = 1,2,3,...$ The DEWMA statistic $W_{t}$​ is constructed in two smoothing stages. The DEWMA charting statistics are defined as:

5 $$\left. \begin{aligned} Z_{t} = \upsilon Y_{t} + (1 - \upsilon )Z_{(t - 1)} \hfill \\ W_{t} = \upsilon Z_{t} + (1 - \upsilon )W_{(t - 1)} \hfill \\ \end{aligned} \right\}$$

where $Z_{0}$ and $W_{0}$ are initialized with the target mean ​$\mu_{0}$(or the in-control process mean) and $W_{t}$ is the DEWMA statistic at time *t.*

Listed below are the DEWMA statistic mean and variance:

$$\begin{aligned} & E(W_{t} ) = \mu_{w} = \mu_{0} \;{\text{and}} \hfill \\ & V(W_{t} ) = \frac{{\upsilon^{4} [1 + (1 - \upsilon )^{2} - (t^{2} + 2t + 1)(1 - \upsilon )^{2t} + (2t^{2} + 2t - 1)(1 - \upsilon )^{2t + 2} - t^{2} (1 - \upsilon )^{2t + 4} ]\sigma_{0}^{2} }}{{(1 - (1 - \upsilon )^{2} )^{3} }}. \hfill \\ \end{aligned}$$

The statistic W_t_ is plotted against the sample number (or time) to construct the DEWMA control chart. The center line and control limits of the DEWMA control chart are defined as follows:

6 $$\left. \begin{aligned} LCL_{t} = \mu_{0} - L\sigma_{0} \sqrt {\frac{{\upsilon^{4} [1 + (1 - \upsilon )^{2} - (t^{2} + 2t + 1)(1 - \upsilon )^{2t} + (2t^{2} + 2t - 1)(1 - \upsilon )^{2t + 2} - t^{2} (1 - \upsilon )^{2t + 4} ]}}{{(1 - (1 - \upsilon )^{2} )^{3} }}} \hfill \\ CL = \mu_{0} \hfill \\ UCL_{t} = \mu_{0} + L\sigma_{0} \sqrt {\frac{{\upsilon^{4} [1 + (1 - \upsilon )^{2} - (t^{2} + 2t + 1)(1 - \upsilon )^{2t} + (2t^{2} + 2t - 1)(1 - \upsilon )^{2t + 2} - t^{2} (1 - \upsilon )^{2t + 4} ]}}{{(1 - (1 - \upsilon )^{2} )^{3} }}} \hfill \\ \end{aligned} \right\}$$

Letting *t* approach infinity will provide the asymptotic variance from the exact variance defined in above equation. So

$$V(W_{t} ) = \frac{{\upsilon (2 - 2\upsilon + \upsilon^{2} )\sigma_{0}^{2} }}{{(2 - \upsilon )^{3} }}.$$

The DEWMA control limits using asymptotic variance are given by

7 $$\left. \begin{aligned} LCL = \mu_{0} - L\sigma_{0} \sqrt {\frac{{\upsilon (2 - 2\upsilon + \upsilon^{2} )}}{{(2 - \upsilon )^{3} }}} \hfill \\ CL = \mu_{0} \hfill \\ UCL = \mu_{0} + L\sigma_{0} \sqrt {\frac{{\upsilon (2 - 2\upsilon + \upsilon^{2} )}}{{(2 - \upsilon )^{3} }}} \hfill \\ \end{aligned} \right\}$$

## The proposed MR DEWMA control chart

A second exponential weighting increases memory and stability by further smoothing the EWMA statistics, enhancing sensitivity to small, sustained shifts while reducing noise and false alarms in process monitoring. Let $V_{t}$ an auxiliary variable, and $U_{t}$, the variable of interest is correlation between the study and auxiliary variable. For each sample, the observations of $U$ and $V$ are obtained in paired from, and it is assumed that the population mean and variance of $V$ are known $\left( {\mu_{v} ,\sigma_{v}^{2} } \right)$. We also suppose that the variables $U$ and $V$ have bivariate normality i.e., $\left( {U,V} \right)\sim N_{1} \left( {\mu_{u} ,\mu_{v} ,\sigma_{u}^{2} ,\sigma_{v}^{2} ,\rho_{uv} } \right)$ where $N_{1}$ shows bivariate normal distribution. Given below is the population mean $\mu_{u}$ Cochran^9^ regression estimate.

8 $$M_{R} = \overline{U} + b_{UV} (\mu_{V} - \overline{V} )$$

where *bUV* measures the expected shift in response to a set percentage increase in the predictor.

The mean and variance are defined as:

9 $$E(M_{R} ) = \mu_{U} ;\;V(M_{R} ) = \sigma_{M}^{2} = \frac{{\sigma_{U}^{2} }}{n}(1 - \rho_{UV}^{2} ) = \frac{{\sigma_{U}^{2} - b_{UV}^{2} \sigma_{V}^{2} }}{n}$$

The plotting statistic for the proposed MR DEWMA chart is defined as

10 $$\left. \begin{aligned} H_{t} = \upsilon M_{{R_{t} }} + (1 - \upsilon )H_{(t - 1)} \hfill \\ C_{t} = \upsilon H_{t} + (1 - \upsilon )C_{(t - 1)} \hfill \\ \end{aligned} \right\}$$

The above equation is based on the regression estimator. Using this information, we have created the M_R_DEWMA control chart. $\upsilon$ Represent the smoothing parameter and M_R_ represent the values from regression estimator for the $tth$ sample. If we put the EWMA-type statistic into double the EWMA statistic, then we have created the M_R_DEWMA control chart.

The mean and variance of the M_R_DEWMA control chart are given below:

$$E(W_{t} ) = \mu_{w} = \mu_{0}$$

$$V(W_{t} ) = \frac{{\upsilon^{4} [1 + (1 - \upsilon )^{2} - (t^{2} + 2t + 1)(1 - \upsilon )^{2t} + (2t^{2} + 2t - 1)(1 - \upsilon )^{2t + 2} - t^{2} (1 - \upsilon )^{2t + 4} ]\sigma_{M}^{2} }}{{(1 - (1 - \upsilon )^{2} )^{3} }}$$

The time-varying control limits for the proposed chart have been established by the M_R_DEWMA statistic which are

11 $$\left. \begin{aligned} LCL_{t} = \mu_{0} - L\sigma_{M} \sqrt {\frac{{\upsilon^{4} [1 + (1 - \upsilon )^{2} - (t^{2} + 2t + 1)(1 - \upsilon )^{2t} + (2t^{2} + 2t - 1)(1 - \upsilon )^{2t + 2} - t^{2} (1 - \upsilon )^{2t + 4} ]}}{{(1 - (1 - \upsilon )^{2} )^{3} }}} \hfill \\ CL = \mu_{0} \hfill \\ UCL_{t} = \mu_{0} + L\sigma_{M} \sqrt {\frac{{\upsilon^{4} [1 + (1 - \upsilon )^{2} - (t^{2} + 2t + 1)(1 - \upsilon )^{2t} + (2t^{2} + 2t - 1)(1 - \upsilon )^{2t + 2} - t^{2} (1 - \upsilon )^{2t + 4} ]}}{{(1 - (1 - \upsilon )^{2} )^{3} }}} \hfill \\ \end{aligned} \right\}$$

Letting $t$ approach infinity will provide the asymptotic variance from the exact variance defined in equation. We have

$$V(W_{t} ) = \frac{{\upsilon (2 - 2\upsilon + \upsilon^{2} )\sigma_{M}^{2} }}{{(2 - \upsilon )^{3} }}.$$

The asymptotic control limits are defined as

12 $$\left. \begin{aligned} LCL = \mu_{0} - L\sigma_{M} \sqrt {\frac{{\upsilon (2 - 2\upsilon + \upsilon^{2} )}}{{(2 - \upsilon )^{3} }}} \hfill \\ CL = \mu_{0} \hfill \\ UCL = \mu_{0} + L\sigma_{M} \sqrt {\frac{{\upsilon (2 - 2\upsilon + \upsilon^{2} )}}{{(2 - \upsilon )^{3} }}} \hfill \\ \end{aligned} \right\}$$

## Performance evaluation

A control chart’s effectiveness is determined by its ability to promptly detect out-of-control (OOC) conditions. This is measured in terms of its RL distribution characteristics. It is defined as the average number of observations before the first OOC point, i.e.

13 $$RL = \inf \{ t \ge 1|C_{t} \ge UCLorC_{t} \le LCL\}$$

The ARL represents the expected number of charting statistics before an out-of-control signal is triggered and serves as a standard performance metric for control charts. Additionally, the SDRL and the MRL are employed to analyze the distribution of run lengths. The proposed control chart’s statistical design required that the combination $(\upsilon ,L)$ have a predefined value of $ARL_{0}$ for example $200,370,500$, etc. SDRL captures the variability in detection time, ensuring consistency, while MRL offers a robust central tendency measure, making both essential for assessing reliability and skewness in run-length distribution.

## Simulation study

In this study, the run-length properties of the M_R_DEWMA control chart are evaluated through Monte Carlo simulations implemented in R statistical software. The simulation algorithm is presented as follows:

Step 1: Generating $10,0000$ random numbers $M_{{R_{t} }}$, where $t$ ranges from 1 to $10,0000$, drawn from a standard normal distribution with parameters $\left( {\mu ,\sigma } \right)$.
Step 2: For a predetermined value of $ARL_{0}$, define the smoothing parameters $\upsilon$ and $L$.
Step 3: Determine the $C_{t}$ statistic by applying Eq. (11).
Step 4: Determine the control limits as specified in Eq. (13) or (15), and then compare each statistic $C_{t}$ against these limits.
Step 5: Monitor the count of charting statistics until the first one exceeds or meets the control limits.
Step 6: The procedure described in steps 1 to 5 is carried out $50,000$ times, and the ARL, MRL, and SDRL are determined by calculating the mean, median, and standard deviation of these $50,000$ outcomes.

The ARL_0_ is calculated using the simulation algorithm mentioned above, with $\mu$ set to $\mu_{0} = 0$ and $\sigma_{0} = 1$ in Step 1. Furthermore, the ARL_1_ is determined for $\mu = \mu_{1} \ne \mu_{0}$. In our simulation study, at $n = 1$, $\upsilon \in \left\{ {0.05, \, 0.1, \, 0.25, \, 0.50, \, 0.75, \, 0.9} \right\}$ and $\rho \in \left\{ {0.05, \, 0.25, \, 0.50, \, 0.75, \, 0.90} \right\}$ for $ARL_{0} = 200$.

Tables 1, 2, 3, 4, 5, 6, 7, 8 and Supplementary tables 9, 10, 11, 12 present the performance comparison of time-varying and asymptotic DEWMA and MRDEWMA control charts based on ARL, SDRL, and MRL profiles, along with their respective control limits.

**Table 1:** ARL, SDRL and MRL values for the DEWMA Control Charts with time-varying control limits.

L	1.71			1.992			2.392			2.678			2.789			2.806		
$\upsilon$	0.05			0.1			0.25			0.5			0.75			0.9		
Shift	ARL	SDRL	MRL	ARL	SDRL	MRL	ARL	SDRL	MRL	ARL	SDRL	MRL	ARL	SDRL	MRL	ARL	SDRL	MRL
0	201.8	246.6	118	200.86	218.98	132	200.31	202.30	140	201.22	202.28	138.5	201.73	203.37	138.5	201.05	202.14	139
0.25	42.113	42.294	31	50.355	46.853	38	67.865	66.918	48	95.240	93.766	67	128.22	128.47	90	144.13	145.79	100
0.5	14.742	13.555	11	17.060	14.156	14	22.852	19.760	17	34.403	31.822	25	54.487	52.781	38	70.93	71.554	49
0.75	7.739	6.7836	6	8.9964	7.0761	7	10.978	8.3106	9	15.17	13.102	12	24.742	23.058	18	36.987	35.328	26
1	4.7352	4.0594	3	5.6352	4.2776	5	6.7674	4.6314	6	8.5416	6.4759	7	12.938	11.527	10	19.420	18.215	14
1.25	3.2884	2.6705	2	3.8568	2.8595	3	4.7072	3.0407	4	5.5214	3.8065	5	7.715	6.3458	6	11.302	10.544	8
1.5	2.4572	1.8363	2	2.8874	2.0276	2	3.5116	2.1379	3	3.964	2.5715	3	5.181	3.9592	4	7.2952	6.5259	5
1.75	1.9582	1.3334	1	2.275	1.4977	2	2.7288	1.645	2	3.0666	1.7814	3	3.7394	2.6427	3	4.9642	4.0755	4
2	1.6278	0.9995	1	1.8686	1.1408	1	2.269	1.2656	2	2.504	1.3699	2	2.8964	1.8611	2	3.6172	2.776	3
2.25	1.4112	0.753	1	1.5868	0.8849	1	1.8934	1.0144	2	2.1046	1.0736	2	2.3628	1.3771	2	2.781	1.9991	2
2.5	1.266	0.5756	1	1.3956	0.6917	1	1.6198	0.8135	1	1.8178	0.8843	2	1.981	1.0545	2	2.2698	1.5002	2
2.75	1.1672	0.4387	1	1.2664	0.5487	1	1.4458	0.6675	1	1.5942	0.7275	1	1.7104	0.8527	2	1.8274	1.0699	2
3	1.1043	0.3402	1	1.1696	0.4272	1	1.3052	0.5453	1	1.4378	0.6195	1	1.5132	0.6936	1	1.5918	0.8579	1

**Table 2:** ARL, SDRL and MRL values for the M_R_ DEWMA Control Charts with time-varying control limits ρ_UV_ = 0.25.

L	1.71			1.992			2.392			2.678			2.789			2.806		
$\upsilon$	0.05			0.1			0.25			0.5			0.75			0.9		
Shift	ARL	SDRL	MRL	ARL	SDRL	MRL	ARL	SDRL	MRL	ARL	SDRL	MRL	ARL	SDRL	MRL	ARL	SDRL	MRL
0	200.65	244.078	116	200.36	221.09	129	200.06	201.03	139	199.99	195.31	138	201.25	204.00	140	199.94	192.61	141
0.25	38.85	39.63	27	46.19	43.85	34	64.92	61.72	45	91.19	91.09	63.5	119.24	119.15	82	137.34	137.7	96
0.5	13.807	12.701	10	16.199	13.767	13	20.800	17.579	16	31.625	29.623	22	50.764	50.964	35	68.715	67.379	48
0.75	7.049	6.379	5	8.418	6.724	7	10.179	7.563	8	13.941	11.869	11	22.633	21.125	16	34.807	34.565	24
1	4.4564	3.8099	3	5.3046	4.0189	4	6.1848	4.2755	5	7.9366	6.172	6	12.0332	10.7836	9	18.1114	17.4425	13
1.25	3.0996	2.5145	2	3.633	2.638	3	4.444	2.8367	4	5.194	3.625	4	7.1372	5.9944	5	10.5368	9.7803	8
1.5	2.269	1.6594	2	2.714	1.908	2	3.3302	2.0719	3	3.694	2.357	3	4.7598	3.5449	4	6.6712	5.8878	5
1.75	1.8256	1.2257	1	2.1474	1.3927	2	2.5848	1.5338	2	2.926	1.6998	3	3.4572	2.3002	3	4.4942	3.6108	3
2	1.5584	0.9342	1	1.7972	1.055	1	2.1066	1.1725	2	2.344	1.2427	2	2.6764	1.6435	2	3.2964	2.5321	3
2.25	1.3574	0.6831	1	1.523	0.821	1	1.8078	0.9289	2	1.972	1.005	2	2.1908	1.2413	2	2.5554	1.7607	2
2.5	1.2282	0.524	1	1.35	0.644	1	1.5592	0.7622	1	1.711	0.8104	2	1.8584	0.9677	2	2.058	1.2925	2
2.75	1.1302	0.4007	1	1.225	0.493	1	1.3984	0.6335	1	1.5268	0.6839	1	1.6014	0.7753	1	1.7176	0.96914	1
3	1.0828	0.3025	1	1.143	0.391	1	1.2716	0.5043	1	1.3842	0.5736	1	1.4486	0.6538	1	1.5074	0.7967	1

**Table 3:** ARL, SDRL and MRL values for the M_R_ DEWMA Control Charts with time-varying control limits ρ_UV_ = 0.50.

L	1.71			1.992			2.392			2.678			2.789			2.806		
$\upsilon$	0.05			0.1			0.25			0.5			0.75			0.9		
Shift	ARL	SDRL	MRL	ARL	SDRL	MRL	ARL	SDRL	MRL	ARL	SDRL	MRL	ARL	SDRL	MRL	ARL	SDRL	MRL
0	200.66	242.03	115	201.19	220.93	133	200.19	201.16	138	200.14	202.11	137	200.71	199.68	142	200.29	199.29	140
0.25	33.282	33.479	24	39.665	37.725	30	54.693	51.725	39	81.19	81.48	57	109.519	108.059	77	127.89	127.16	90
0.5	11.686	10.713	9	13.72	11.049	11	17.0054	14.1699	13	25.9	24.12	18	40.3	39.56	29	58.98	56.75	41
0.75	6.0684	5.3639	5	7.2	5.54	6	8.535	6.1793	7	11.24	9.15	9	18.08	17.66	13	27.01	25.76	20
1	3.729	3.123	3	4.397	3.36	3	5.2928	3.4815	5	6.38	4.68	5	9.304	8.06	7	14.124	13.46	10
1.25	2.593	2.023	2	3.06	2.144	2	3.7034	2.2791	3	4.28	2.73	4	5.52	4.216	4	8.08	7.204	6
1.5	1.9532	1.3499	1	2.31	1.54	2	2.7602	1.6384	2	3.079	1.78	3	3.78	2.702	3	5.102	4.177	4
1.75	1.5944	0.9491	1	1.84	1.128	1	2.1728	1.2287	2	2.435	1.311	2	2.78	1.78	2	3.501	2.69	3
2	1.3644	0.6954	1	1.538	0.845	1	1.8138	0.96525	2	2.01	1.012	2	2.25	1.294	2	2.589	1.744	2
2.25	1.2258	0.5185	1	1.343	0.62	1	1.555	0.7644	1	1.714	0.814	2	1.84	0.95	2	2.0198	1.254	2
2.5	1.1252	0.3659	1	1.214	0.488	1	1.3788	0.6143	1	1.493	0.66	1	1.583	0.742	1	1.677	0.923	1
2.75	1.0742	0.2834	1	1.122	0.363	1	1.2428	0.4811	1	1.34	0.539	1	1.397	0.609	1	1.46	0.716	1
3	1.043	0.2102	1	1.079	0.284	1	1.152	0.3854	1	1.22	0.45	1	1.27	0.497	1	1.293	0.546	1

**Table 4:** ARL, SDRL and MRL values for the M_R_ DEWMA Control Charts with time-varying control limits ρ_UV_ = 0.75.

L	1.71			1.992			2.392			2.678			2.789			2.806		
$\upsilon$	0.05			0.1			0.25			0.5			0.75			0.9		
Shift	ARL	SDRL	MRL	ARL	SDRL	MRL	ARL	SDRL	MRL	ARL	SDRL	MRL	ARL	SDRL	MRL	ARL	SDRL	MRL
0	201.09	247.49	117	200.8	216.16	131	201.19	208.17	138	200.89	198.12	141	200.37	204.37	140	200.53	199.43	139.5
0.25	22.66	22.14	17	27.03	24.03	21	36.2998	34.4399	26	55.03	52.73	39	80.08	80.77	55	102.7	103.02	71
0.5	7.556	6.707	6	8.902	6.914	7	10.5496	8.02	9	14.838	12.538	11	23.907	22.789	17	36.18	34.74	26
0.75	3.8906	3.235	3	4.608	3.466	4	5.4024	3.5804	5	6.73	4.972	5	9.532	8.056	7	14.28	13.75	10
1	2.4452	1.841	2	2.859	1.996	2	3.448	2.1581	3	3.8996	2.399	3	5.053	3.876	4	7.22	6.27	5
1.25	1.7582	1.1322	1	2.037	1.2796	2	2.4016	1.3892	2	2.7412	1.502	2	3.2426	2.176	3	4.087	3.254	3
1.5	1.3992	0.741	1	1.5816	0.882	1	1.844	0.9945	2	2.078	1.055	2	2.3068	1.345	2	2.706	1.912	2
1.75	1.205	0.4768	1	1.3194	0.6228	1	1.53	0.73403	1	1.6808	0.775	2	1.807	0.924	2	2.024	1.269	2
2	1.102	0.3359	1	1.177	0.4295	1	1.296	0.5284	1	1.4172	0.596	1	1.494	0.686	1	1.589	0.857	1
2.25	1.0504	0.2215	1	1.0788	0.286	1	1.167	0.40093	1	1.244	0.467	1	1.288	0.509	1	1.33	0.596	1
2.5	1.0178	0.1352	1	1.0342	0.185	1	1.0856	0.2896	1	1.145	0.3624	1	1.1692	0.394	1	1.188	0.441	1
2.75	1.0092	0.0954	1	1.0134	0.1149	1	1.0374	0.19081	1	1.072	0.262	1	1.093	0.295	1	1.09	0.2997	1
3	1.0018	0.0469	1	1.0056	0.0746	1	1.0166	0.1278	1	1.029	0.168	1	1.044	0.2056	1	1.043	0.2034	1

**Table 5:** ARL, SDRL and MRL values for the DEWMA Control Charts with asymptotic control limits.

L	1.71			1.992			2.392			2.678			2.789			2.806		
$\upsilon$	0.05			0.1			0.25			0.5			0.75			0.9		
Shift	ARL	SDRL	MRL	ARL	SDRL	MRL	ARL	SDRL	MRL	ARL	SDRL	MRL	ARL	SDRL	MRL	ARL	SDRL	MRL
0	201.08	181.85	146	201.02	192.69	141.5	200.96	200.73	139	200.21	197.47	138	200.14	198.08	139	200.39	198.64	143
0.25	54.515	35.373	44	57.464	44.014	45	70.74	64.182	51	96.495	92.961	67	128.64	127.51	90	147.42	147.23	101
0.5	26.337	10.014	24	22.918	12.075	20	24.674	19.108	19	34.761	31.747	25	54.725	53.254	38	73.341	71.318	52
0.75	18.801	4.857	18	14.77	5.460	13	12.742	7.476	11	15.883	13.241	12	25.432	24.054	18	37.981	36.747	26
1	15.169	3.058	15	11.315	3.0477	11	8.536	3.9962	7	9.0862	6.524	7	13.409	11.515	10	20.263	19.371	14
1.25	13.039	2.1047	13	9.4504	2.0039	9	6.5526	2.4873	6	6.1316	3.6596	5	8.0714	6.5139	6	11.708	10.771	8
1.5	11.559	1.6223	11	8.2706	1.5156	8	5.448	1.6632	5	4.5618	2.3596	4	5.4514	4.0347	4	7.452	6.6559	5
1.75	10.495	1.3437	10	7.4006	1.2003	7	4.7026	1.2077	5	3.6714	1.5758	3	3.9848	2.6812	3	5.042	4.1388	4
2	9.6664	1.0850	10	6.7838	1.0008	7	4.2052	0.9729	4	3.0954	1.1806	3	3.1054	1.8255	3	3.7084	2.8305	3
2.25	9.0058	0.9438	9	6.2797	0.8451	6	3.8168	0.7859	4	2.7376	0.8991	3	2.5066	1.3065	2	2.8346	2.0012	2
2.5	8.447	0.8434	8	5.8686	0.738	6	3.5338	0.6809	3	2.4728	0.7173	2	2.1638	1.0317	2	2.3046	1.461	2
2.75	7.9728	0.7308	8	5.532	0.6523	5	3.297	0.5693	3	2.2742	0.5854	2	1.902	0.8276	2	1.912	1.1157	2
3	7.5878	0.6606	8	5.2268	0.5749	5	3.1176	0.4881	3	2.1236	0.4878	2	1.701	0.7077	2	1.6332	0.8887	1

**Table 6:** ARL, SDRL and MRL values for the M_R_ DEWMA Control Charts with asymptotic control limits ρ_UV_ = 0.25.

L	1.71			1.992			2.392			2.678			2.789			2.806		
$\upsilon$	0.05			0.1			0.25			0.5			0.75			0.9		
Shift	ARL	SDRL	MRL	ARL	SDRL	MRL	ARL	SDRL	MRL	ARL	SDRL	MRL	ARL	SDRL	MRL	ARL	SDRL	MRL
0	200.30	182.09	143	200.76	186.15	144	200.29	188.09	144	199.60	201.22	139	201.25	200.9	138	199.97	198.99	137
0.25	51.984	33.516	42	53.209	40.274	41	67.799	62.303	49	92.53	86.846	67	122.75	122.47	84.5	144.31	139.23	102
0.5	25.399	9.2897	23	21.765	10.979	19	22.472	16.796	17	32.104	29.083	24	51.220	50.206	36	69.977	68.605	49
0.75	18.071	4.4605	17	14.160	4.8025	13	12.06	6.9835	10	14.805	11.758	11	23.296	22.072	17	35.128	34.424	25
1	14.682	2.7822	14	10.89	2.8137	10	8.109	3.6365	7	8.543	5.9159	7	12.323	11.057	9	18.43	17.652	13
1.25	12.680	1.9801	12	9.157	1.9142	9	6.288	2.2269	6	5.778	3.4915	5	7.437	5.8491	6	10.75	9.7973	8
1.5	11.284	1.536	11	8.0294	1.42075	8	5.204	1.4996	5	4.3382	2.10369	4	5.0984	3.67911	4	6.749	5.8088	5
1.75	10.24	1.2418	10	7.2094	1.13834	7	4.5242	1.13158	4	3.5244	1.50089	3	3.6772	2.2687	3	4.6612	3.72306	4
2	9.443	1.0381	9	6.588	0.92802	6	4.0616	0.8852	4	2.9796	1.06799	3	2.894	1.6616	2	3.3846	2.51609	2
2.25	8.818	0.8899	9	6.122	0.79652	6	3.7148	0.76235	4	2.6204	0.80525	2	2.3896	1.2212	2	2.5958	1.7277	2
2.5	8.265	0.7778	8	5.7098	0.7046	6	3.4368	0.6242	3	2.3698	0.6468	2	2.0588	0.9412	2	2.1342	1.31626	2
2.75	7.812	0.7030	8	5.3978	0.6239	5	3.2116	0.52429	3	2.1892	0.52446	2	1.7976	0.76678	2	1.778	1.00664	2
3	7.455	0.6409	7	5.1372	0.5521	5	3.0458	0.4814	3	2.0648	0.4663	2	1.6188	0.6597	2	1.533	0.7768	1

**Table 7:** ARL, SDRL and MRL values for the M_R_ DEWMA Control Charts with asymptotic control limits ρ_UV_ = 0.50.

L	1.71			1.992			2.392			2.678			2.789			2.806		
$\upsilon$	0.05			0.1			0.25			0.5			0.75			0.9		
Shift	ARL	SDRL	MRL	ARL	SDRL	MRL	ARL	SDRL	MRL	ARL	SDRL	MRL	ARL	SDRL	MRL	ARL	SDRL	MRL
0	200.05	175.31	147	200.13	188.76	145	200.19	196.28	140	200.89	203.87	139	201.24	199.47	140	200.37	198.26	141
0.25	45.89	26.673	38	46.624	34.782	37	56.366	50.012	41	81.709	77.869	59	112.55	110.72	77	131.76	131.29	93
0.5	23.07	7.4447	22	19.535	9.0226	17	19.159	13.880	15	26.411	24.287	19	42.196	41.364	30	59.355	57.924	42
0.75	16.68	3.6557	16	12.763	3.9472	12	10.37	5.4511	9	11.92	9.3485	9	18.55	17.39	13	27.697	26.386	20
1	13.65	2.3358	13	10.06	2.4439	10	7.197	2.8734	7	6.999	4.5026	6	9.619	8.124	7	14.17	13.038	10
1.25	11.84	1.7003	12	8.449	1.6204	8	5.566	1.7427	5	4.763	2.5489	4	5.924	4.469	5	8.163	7.2291	6
1.5	10.53	1.3276	10	7.448	1.2335	7	4.713	1.2004	5	3.731	1.6401	3	4.064	2.673	3	5.314	4.4468	4
1.75	9.597	1.0807	9	6.726	0.9599	7	4.178	0.9534	4	3.053	1.1517	3	3.083	1.747	3	3.606	2.764	3
2	8.834	0.8976	9	6.166	0.8229	6	3.729	0.7495	4	2.643	0.8300	2	2.433	1.245	2	2.710	1.857	2
2.25	8.274	0.7892	8	5.727	0.711	6	3.427	0.6190	3	2.364	0.6573	2	2.049	0.942	2	2.120	1.312	2
2.5	7.744	0.6959	8	5.349	0.6087	5	3.1928	0.5209	3	2.1812	0.51847	2	1.759	0.7488	2	1.7392	0.9661	1
2.75	7.356	0.6127	7	5.0578	0.5467	5	3.0078	0.46796	3	2.024	0.4524	2	1.5634	0.6369	1	1.5028	0.7414	1
3	6.992	0.5627	7	4.8066	0.5296	5	2.8486	0.4588	3	1.9232	0.4115	2	1.4058	0.5426	1	1.3254	0.5657	1

**Table 8:** ARL, SDRL and MRL values for the M_R_ DEWMA Control Charts with asymptotic control limits ρ_UV_ = 0.75.

L	1.71			1.992			2.392			2.678			2.789			2.806		
$\upsilon$	0.05			0.1			0.25			0.5			0.75			0.9		
Shift	ARL	SDRL	MRL	ARL	SDRL	MRL	ARL	SDRL	MRL	ARL	SDRL	MRL	ARL	SDRL	MRL	ARL	SDRL	MRL
0	200.63	180.45	145	200.11	189.72	146	200.04	192.57	143	201.02	196.29	142	200.43	200.95	138	200.99	202.28	139
0.25	34.723	16.67	30	32.4	20.71	26	37.449	30.83	28	55.476	52.829	39	82.024	80.110	56	105.16	103.17	75
0.5	18.495	4.642	18	14.658	5.547	13	12.67	7.560	10	15.53	12.732	12	24.11	22.61	17	37.220	36.246	26
0.75	13.846	2.4326	14	10.121	2.386	10	7.26	2.958	7	7.196	4.6871	6	9.641	7.837	7	14.99	14.44	11
1	11.4396	1.6132	11	8.1784	1.4789	8	5.387	1.6299	5	4.4676	2.2781	4	5.318	3.8938	4	7.2426	6.3334	5
1.25	9.986	1.1821	10	7.0116	1.0521	7	4.3854	1.0409	4	3.2782	1.2789	3	3.421	2.1174	3	4.2124	3.3544	3
1.5	8.9308	0.9142	9	6.2296	0.8511	6	3.767	0.7668	4	2.7176	0.8852	3	2.4946	1.2868	2	2.7498	1.8802	2
1.75	8.166	0.7556	8	5.653	0.6878	6	3.3938	0.6213	3	2.3342	0.62079	2	1.987	0.91	2	2.0254	1.213	2
2	7.5532	0.6499	8	5.2146	0.5715	5	3.0978	0.5058	3	2.1022	0.4773	2	1.675	0.6953	2	1.63	0.8635	1
2.25	7.0708	0.5708	7	4.8542	0.5224	5	2.8836	0.4959	3	1.9524	0.4335	2	1.45	0.5758	1	1.3612	0.6089	1
2.5	6.6596	0.5548	7	4.5334	0.5198	5	2.6708	0.4754	3	1.8182	0.4277	2	1.2782	0.4704	1	1.1964	0.4389	1
2.75	6.2814	0.4786	6	4.258	0.4426	4	2.457	0.4557	2	1.6714	0.4169	2	1.1638	0.3787	1	1.1016	0.3126	1
3	6.0234	0.3683	6	4.0732	0.3031	4	2.2578	0.4375	2	1.5338	0.4017	2	1.086	0.2818	1	1.0474	0.2162	1

## Results and discussion

The following are the main findings of our proposed MRDEWMA control chart for monitoring a process’s location (see Tables 1, 2, 3, 4, 5, 6, 7 and 8).

i. Considering ARL values:
  The performance of the MRDEWMA chart was greatly improved by the addition of auxiliary information through a regression estimator, particularly at higher values of $\rho_{VU}$. For instance, when there is a shift of magnitude 0.5, the existing DEWMA chart that utilizes time-varying control limits identifies the shift at the 15th sample. On the other hand, the proposed MRDEWMA chart spots the same shift earlier, at the 12th sample, with $\rho_{VU} = 0.50$.
  The proposed MRDEWMA chart, which incorporates time-varying control limits, reveals superior performance for smaller values of *v*, with fixed values of $\rho_{VU}$ and the process shift. For example, when $\rho_{VU} = 0.25$ and the shift equals 0.5, the proposed chart finds the OOC point at the 14th sample for a small value of $\upsilon = 0.05$, while for a larger value of $\upsilon = 0.75$, the same OOC point is detected at the 51st sample.
  The proposed MRDEWMA chart is ARL-unbiased, which means that regardless of the shift value, the $ARL_{1}$ does not go beyond the $ARL_{0}$ for any given choice of $\upsilon$, *L*, and $\rho_{VU}$.
  For small values of $\rho_{VU}$, the ARL of the proposed chart reduces progressively with an increase in shift, whereas for larger values of $\rho_{VU}$, the ARL falls quickly as the shift grows for more details see Tables 2, 3, 4).
ii. Considering SDRL values:
  A significant positive association is identified between SDRL values and the smoothing parameter $\upsilon$, as SDRL values elevate alongside the increase in $\upsilon$. For example, when $\rho_{VU} = 0.25$ and the shift is 0.75, the SDRL1 value is 8.418 for a lower value of $\upsilon = 0.1$, while it rises to 34.807 when $\upsilon = 0.9$.
  The SDRL values for the suggested MRDEWMA chart show a declining trend as the value of $\rho_{VU}$ increases, regardless of the value of $\upsilon$. This trend implies a greater chance of false OOC signals with a rise in $\rho_{VU}$. For instance, at $\rho_{VU} = 0.25$ and a process shift of $\upsilon = 0.25$, the SDRL1 value is 64.92. However, when $\rho_{VU}$ is increased to 0.50, the SDRL1 value decreases to 54.693.
  The proposed MRDEWMA chart is SDRL-unbiased, which means that regardless of the shift value, the SDRL1 does not go beyond the SDRL0for any given choice of $\upsilon$, *L*, and $\rho_{VU}$.
iii. Considering MRL values:
  The MRDEWMA chart illustrates enhanced performance when auxiliary variables are taken into account, especially when the value of $\rho_{VU}$ is quite large, as distinctly shown by the MRL1 values.
  The suggested control chart results in reduced MRL values for higher values of $\rho_{VU}$, especially in cases of minor and moderate mean shifts. For instance, the MRDEWMA control chart generates an MRL of 57 when $\rho_{VU} = 0.5$, $\upsilon = 0.5$, and the shift size is 0.25. In contrast, the MRL drops to 39 when $\rho_{VU} = 0.75$, maintaining $\upsilon = 0.5$ and the same shift size.
  Higher values of $\upsilon$ correlate with elevated MRL values, regardless of the $\rho_{VU}$ value or the intended degree of the control chart shift. For instance, when $\rho_{VU} = 0.75$ and $\upsilon = 0.05$, using a shift size of 0.75, the resulting MRL1 is 3.8906. In the same way, when $\upsilon = 0.75$, the MRL1 rises to 9.532.

## Comparisons

This section provides a comprehensive comparison of the proposed M_R_DEWMA chart (time-varying) with the DEWMA chart (time-varying), in addition to comparing the M_R_DEWMA chart (asymptotic) with the M_R_DEWMA chart (time-varying). The aim is to assess the performance of the proposed chart in relation to its current counterparts under both time-varying and asymptotic scenarios.

### Proposed M_R_DEWMA time-varying vs. existing DEWMA time-varying chart

**Fig. 1:**
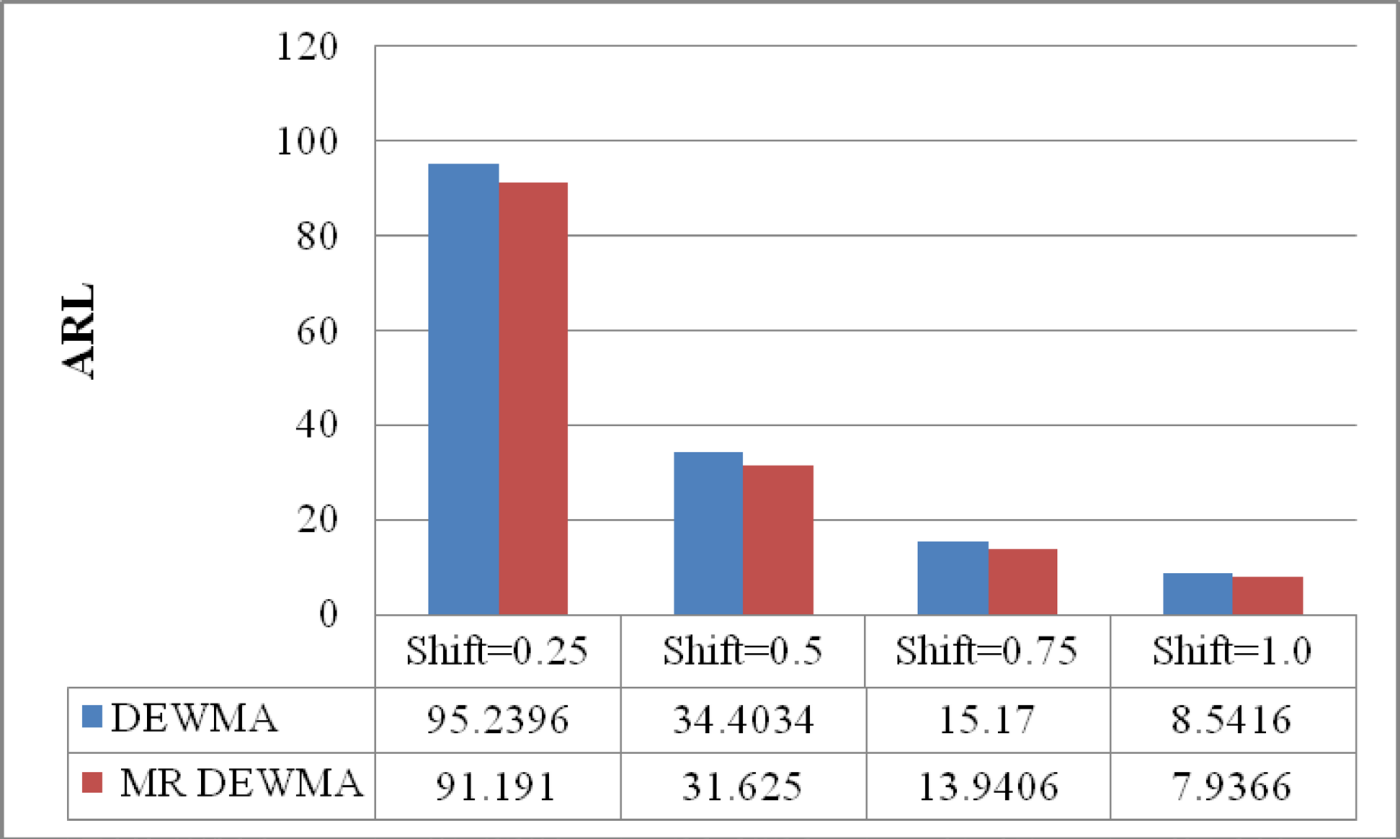
Performance comparison of the time varying DEWMA and M_R_DEWMA in terms of ARL when $\upsilon = 0.50$ and $\rho = 0.25$.

**Fig. 2:**
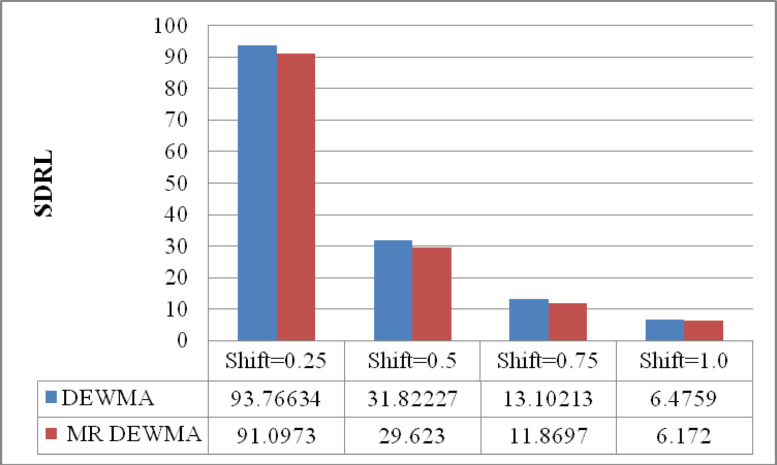
Performance comparison of the time varying DEWMA and M_R_DEWMA in terms of SDRL when $\upsilon = 0.50$ and $\rho = 0.25$.

**Fig. 3:**
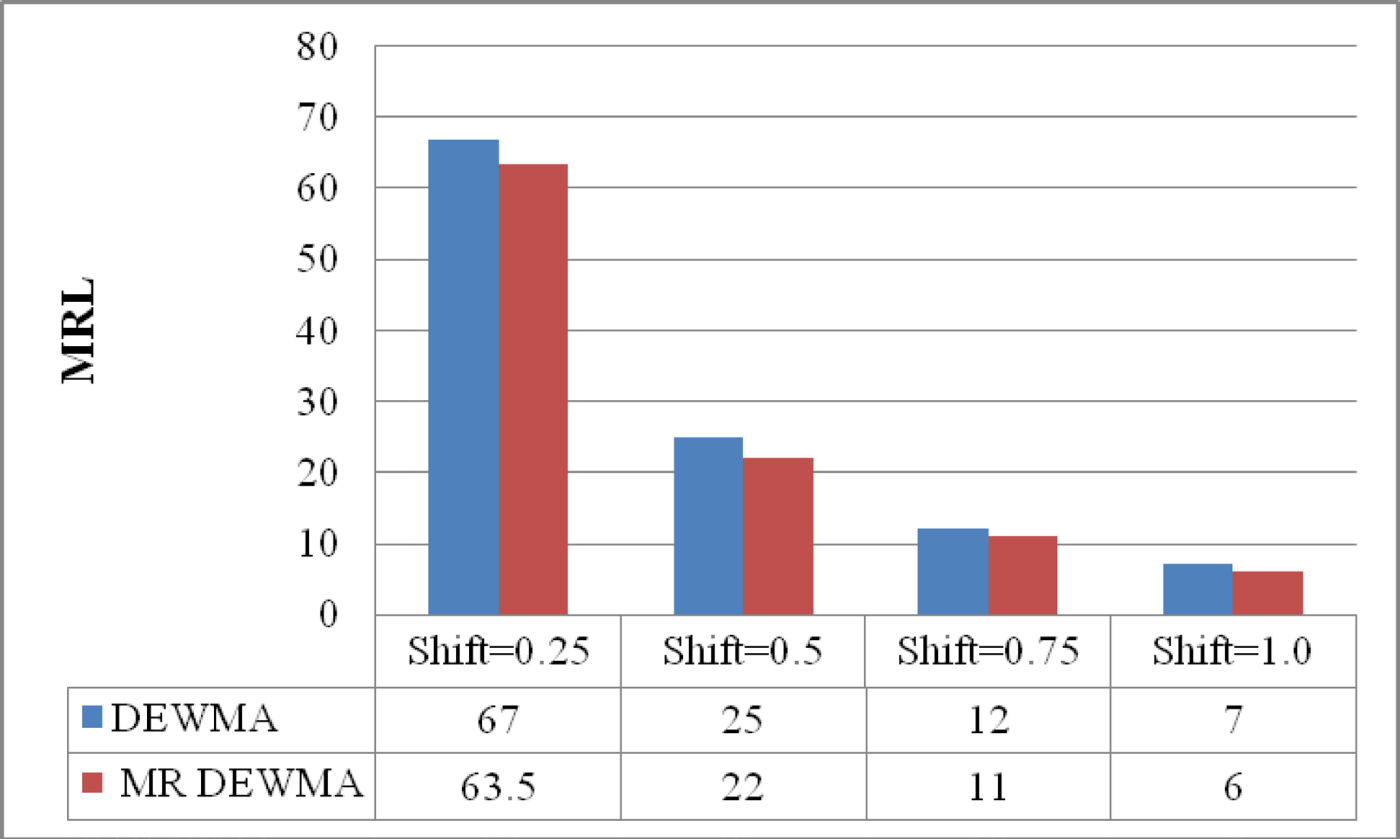
Performance comparison of the time varying DEWMA and M_R_DEWMA in terms of MRL when $\upsilon = 0.50$ and $\rho = 0.25$.

Table 1 provides the ARL, SDRL, and MRL values for the time-varying DEWMA control chart. Figures 1, 2, and 3 presented a comparative analysis of the time-varying DEWMA and M_R_DEWMA schemes, emphasizing their respective ARL, SDRL, and MRL profiles for $n = 1$ and $\upsilon = 0.50$, with $\rho = 0.25$ and a nominal $ARL_{0} = 200$. To effectively showcase the differences between the DEWMA and M_R_DEWMA monitoring schemes under various conditions, all graphs are plotted on a consistent scale.

The data presented in Figs. 1, 2, 3 indicate that the M_R_DEWMA scheme yields more favourable ARL, SDRL, and MRL profiles compared to the DEWMA scheme, particularly for small to moderate shifts in the process mean. However, for larger shifts, both schemes demonstrate comparable performance across these metrics. In short, the M_R_DEWMA scheme offers a distinct advantage over the DEWMA scheme in detecting small to moderate process shifts.

### Effect of correlation on the performance of the proposed M_R_DEWMA chart

**Fig. 4:**
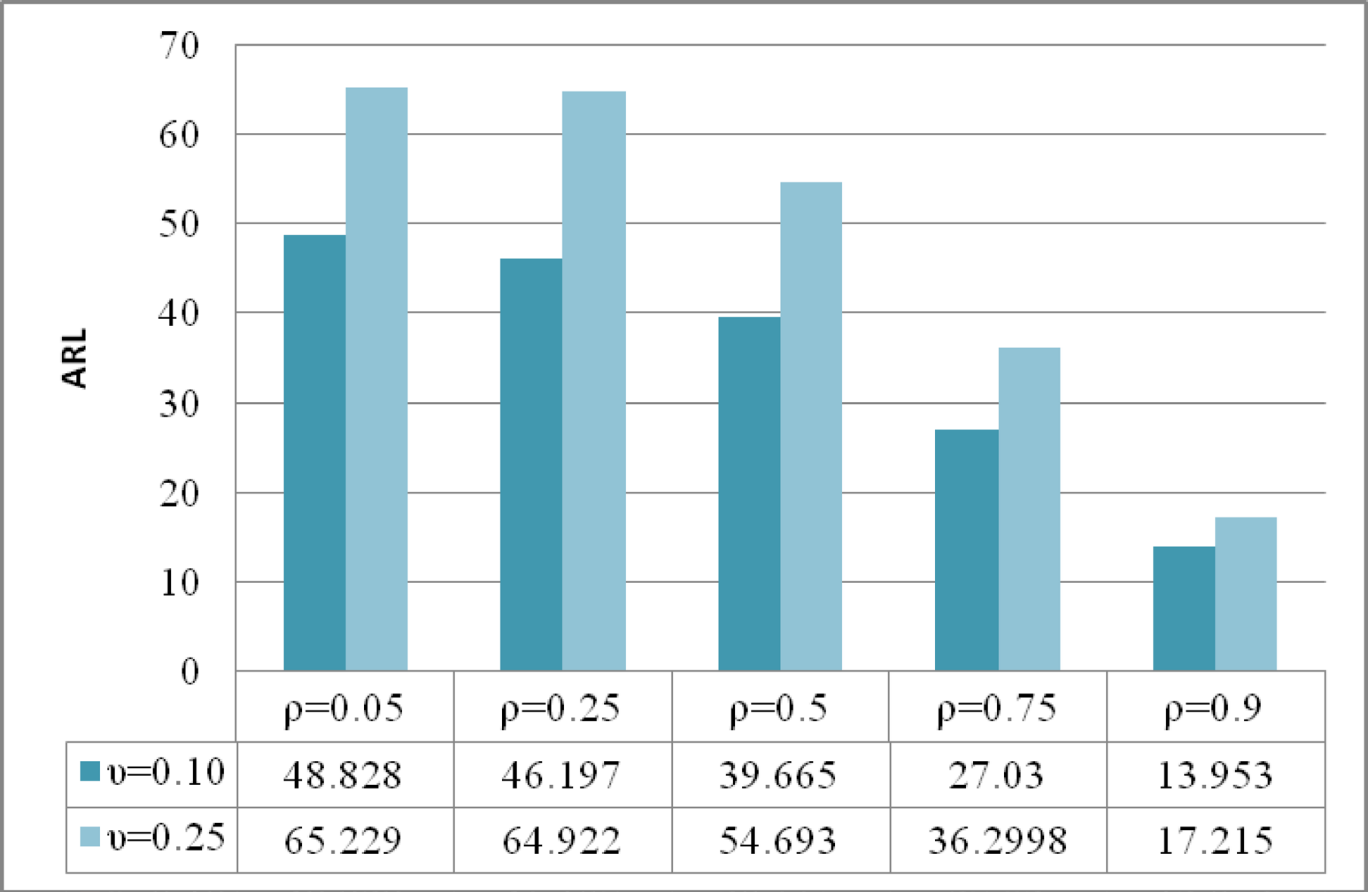
Performance comparison of the time varying M_R_DEWMA in terms of ARL using different correlation levels and smoothing parameters.

**Fig. 5:**
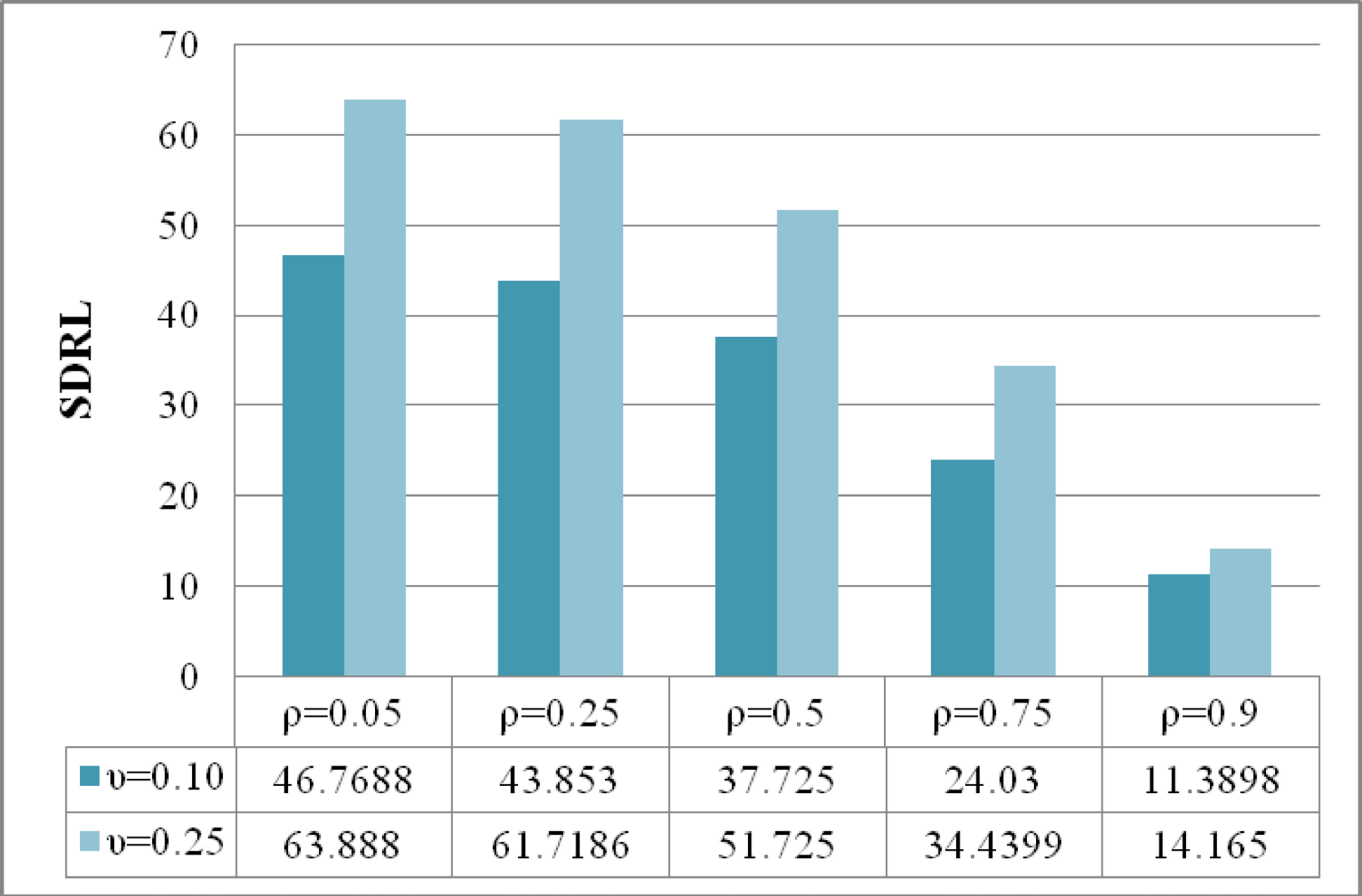
Performance comparison of the time varying M_R_DEWMA in terms of SDRL using different correlation levels and smoothing parameters.

**Fig. 6:**
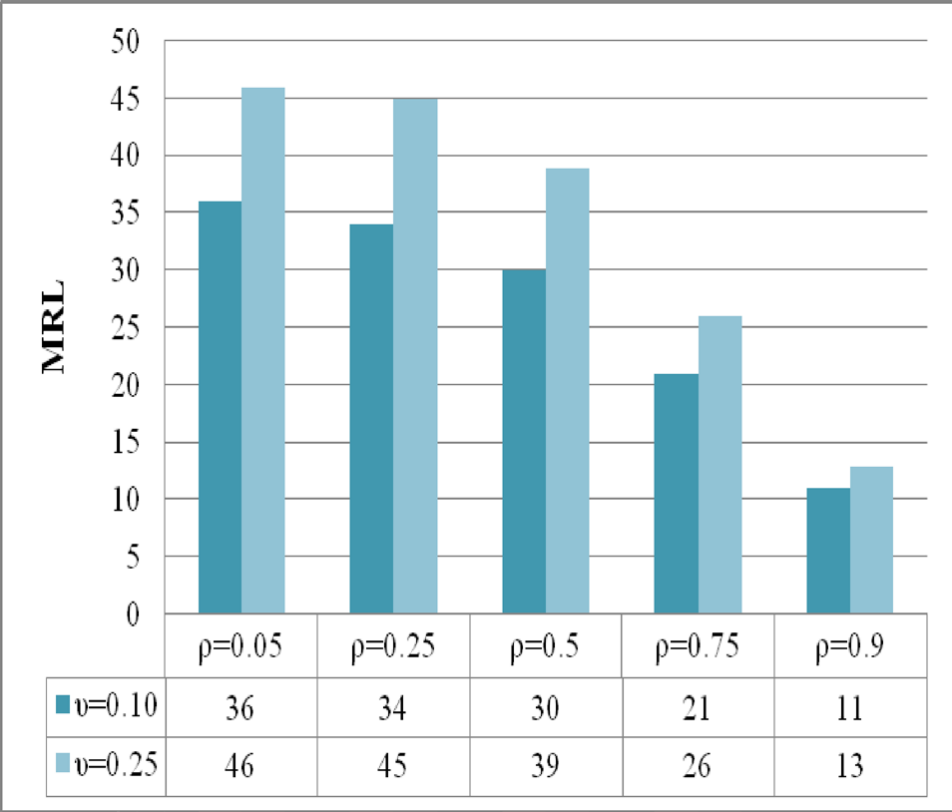
Performance comparison of the time varying M_R_DEWMA in terms of MRL using different correlation levels and smoothing parameters.

The analysis presented in Figs. 4, 5, 6 provides a comprehensive assessment of the efficiency characteristics of the time-varying M_R_DEWMA control chart focusing on three key performance metrics: ARL, SDRL, and MRL. The evaluation is conducted for a sample size of and along with $\rho = \left\{ {0.05,0.25,0.50,0.75,0.9} \right\},$ while maintaining a nominal $ARL_{0} = 200$. The detection capability of the M_R_DEWMA method is enhanced with increased correlation among the variables. This enhancement is reflected in the profiles of ARL, SDRL, and MRL. As the correlation rises, the method demonstrates a more efficient response to process shifts, leading to quicker detection times and more dependable performance metrics. Additionally, the M_R_DEWMA chart demonstrates superior effectiveness when lower values of the smoothing parameter are employed. This is particularly evident in the ARL, SDRL, and MRL metrics, where smaller smoothing parameters significantly enhance the chart’s sensitivity and responsiveness. These findings underscore the importance of appropriately selecting the smoothing parameter to optimize the monitoring and detection efficiency of the M_R_DEWMA method.

It is also noteworthy that the DEWMA chart represents a special case of the M_R_DEWMA chart; specifically, when the smoothing parameters are equal, the M_R_DEWMA reduces to the standard DEWMA chart. Furthermore, as illustrated in Tables 2, 3, 4, 5, and 6, increasing the value of the smoothing parameter consistently improves the performance of the proposed chart.

### Time-varying M_R_DEWMA vs. asymptotic M_R_DEWMA performance

**Fig. 7:**
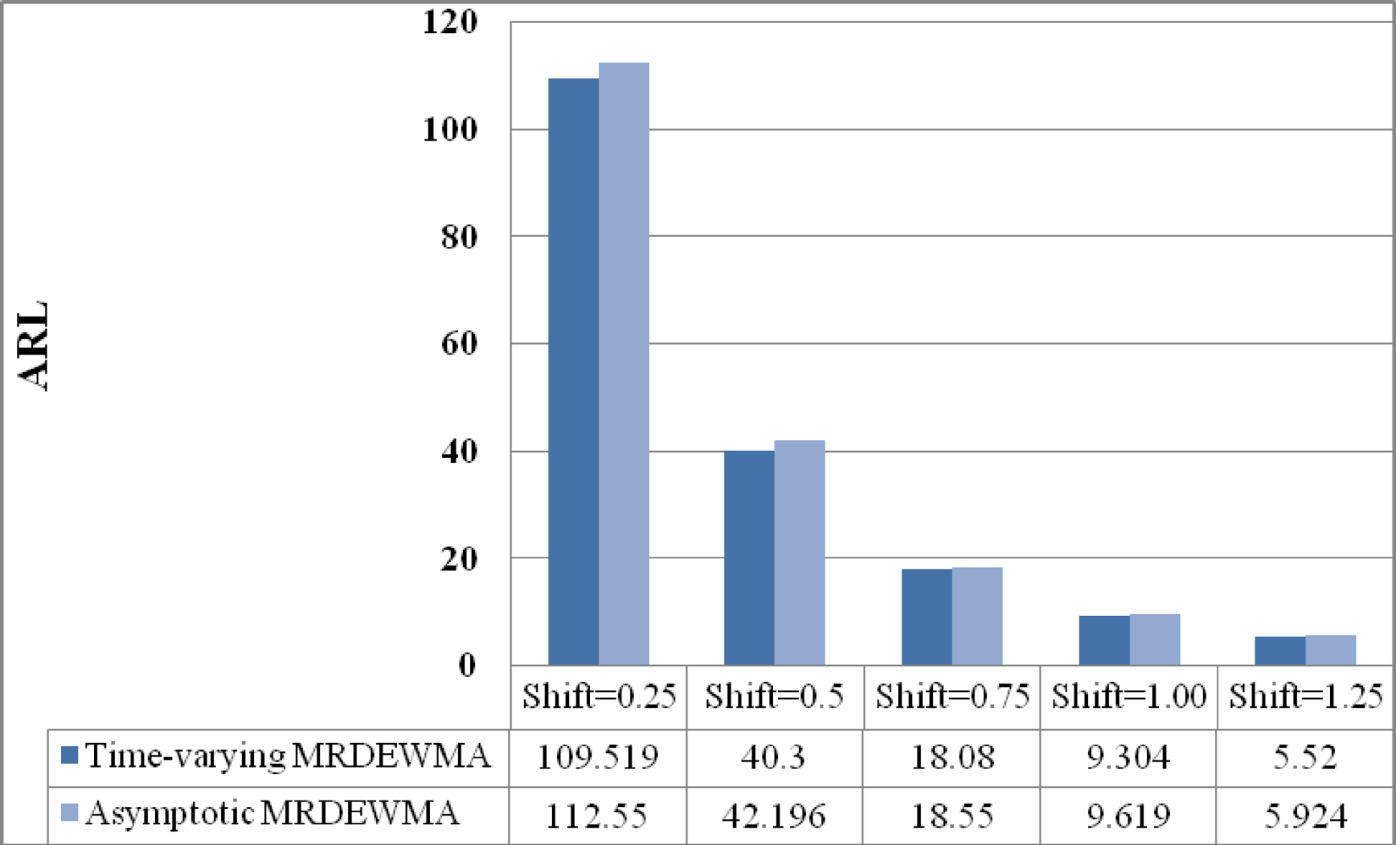
Performance comparison of asymptotic and time-varying M_R_DEWMA chart in terms of ARL.

**Fig. 8:**
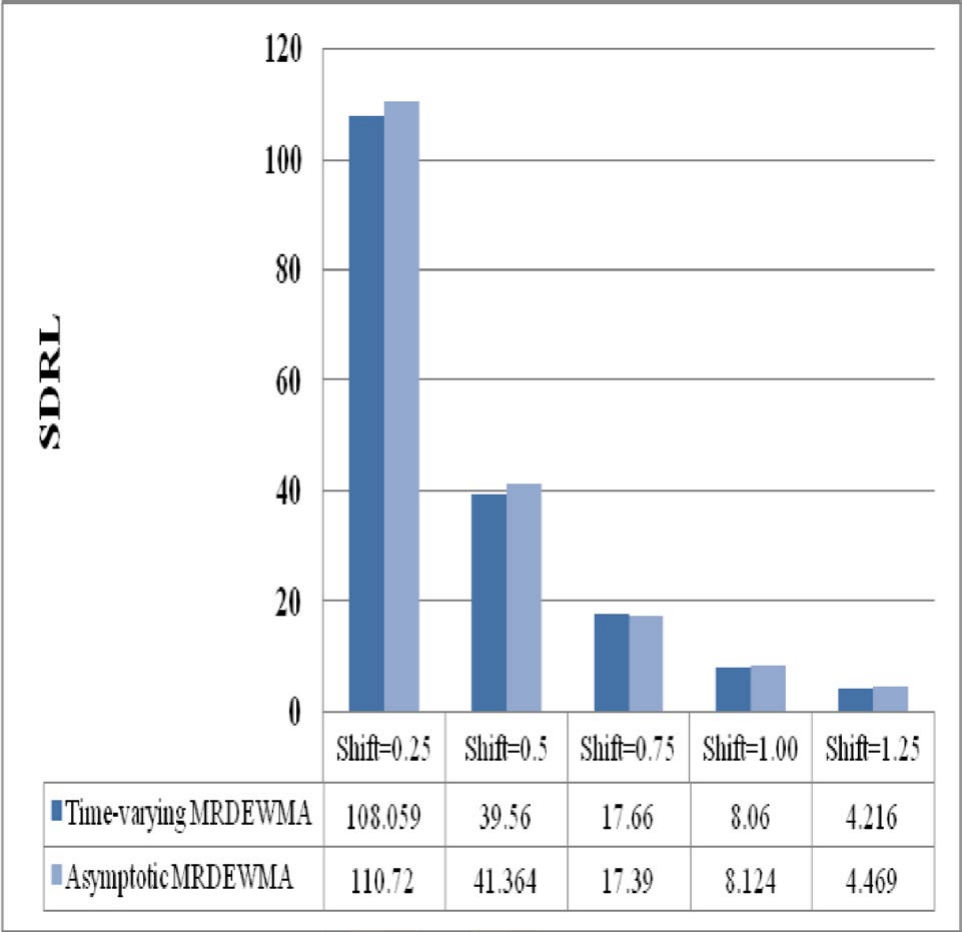
Performance comparison of asymptotic and time-varying M_R_DEWMA chart in terms of SDRL.

**Fig. 9:**
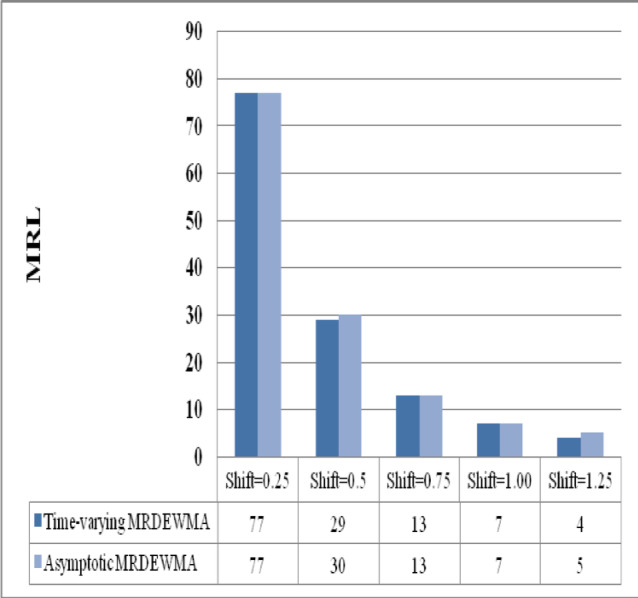
Performance comparison of asymptotic and time-varying M_R_DEWMA chart in terms of MRL.

The asymptotic M_R_DEWMA control chart’s performance when $n = 1$, $\rho \in \left\{ {0.05, \, 0.25, \, 0.50, \, 0.75, \, 0.90} \right\}$, and $\upsilon \in \left( {0.05, \, 0.1, \, 0.25, \, 0.50, \, 0.75, \, 0.9} \right)$ are investigated for a nominal $ARL_{0} = 200$ in Table 8 and Supplemenatry tables 9, 10, 11, 12. It is obvious that the process is operating in a steady state (i.e., over an extended period), the performance of the proposed M_R_DEWMA chart significantly decreases when $\upsilon \in \left( {0, \, 0.75} \right)$. In other words, regardless of the $\rho_{VU}$ value, the M_R_DEWMA chart exhibits poor performance for small and moderate values in the asymptotic case. Figures 7, 8, 9 illustrate that the proposed time-varying MRDEWMA chart outperforms the asymptotic MRDEWMA chart in terms of Average Run Length (ARL) and Standard Deviation of Run Length (SDRL). However, both charts exhibit approximately similar performance with respect to Median Run Length (MRL).

## Case study for the simulated data set

**Fig. 10:**
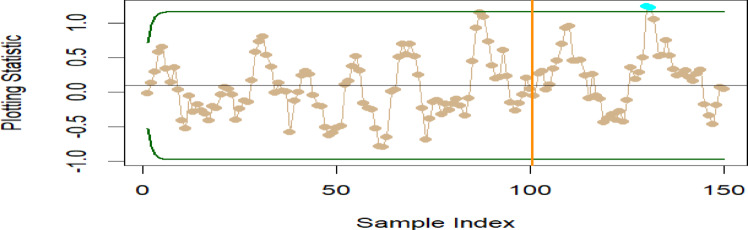
DEWMA chart for the simulated data set.

**Fig. 11:**
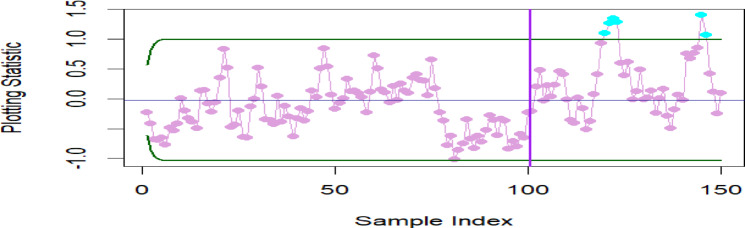
M_R_DEWMA chart for the simulated data set.

To evaluate the effectiveness of the proposed M_R_DEWMA chart in relation to the DEWMA chart under a slight shift in the process mean, we simulated 150 observations from a standard normal distribution $\left( {\mu = 0, \, \sigma^{2} = 1} \right)$. The IC process mean is defined as $\mu_{0} = 0$. Specifically, the first 100 observations are taken from $N(0, \, 1)$, while the last 50 observations are taken from $N(0.25, \, 1)$, which signifies an OOC shift of 0.25. We implemented time-varying control limits, with the objective of achieving an in-control average run length (ARL₀) of 200. Accordingly, the smoothing parameter is set to $\upsilon = 0.50$, and the control limit half-width was established at $L = 2.678$ for both the M_R_DWMA and DEWMA charts. In the case of the M_R_DEWMA chart, the correlation coefficient $\rho_{VU}$ is fixed at 0.50. Figures 10, 11 illustrate the effectiveness of both the proposed and existing charts. The M_R_DWMA chart detects shifts after 119, 120, and 121, etc. observations, while the DEWMA charts identify shifts after 139, 129 observations. This emphasizes the M_R_DWMA chart’s superior sensitivity in detecting minor process shifts compared to the DEWMA chart.

## Real life application

Motivated by Lucas et al.^30^, we employ a real dataset from R Studio comprising 28 observations. The dataset pertains to the tensile strength values of raw materials used in the clothing industry.

For this purpose, we have taken a real dataset from the R studio containing 28 observations. The name of the dataset tensile strength values raw material for the clothing industry. We are evaluating the tensile strength of two distinct shipments of raw materials in the textile industry, which follows an approximately bivariate normal distribution. The mean and variance are given by:

$$\mu_{x} = \left[ {\begin{array}{*{20}c} {68.71} \\ {69.57} \\ \end{array} } \right]\;and\;\sigma_{x}^{2} = \left[ {\begin{array}{*{20}c} {2.04} & {1.6014} \\ {1.6014} & {1.60} \\ \end{array} } \right],$$

**Fig. 12:**
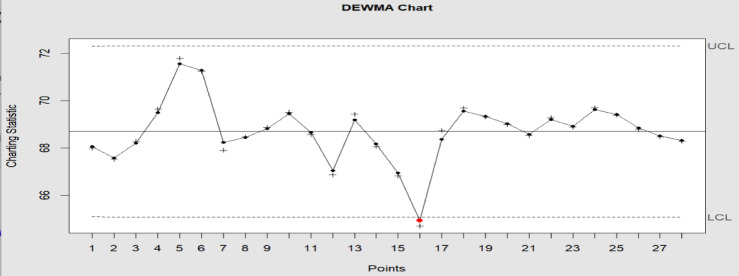
DEWMA control chart with time varying limits, parameters of chart $\upsilon = 0.9$ and $L = 2.806$ at $ARL_{0} = 200$.

**Fig. 13:**
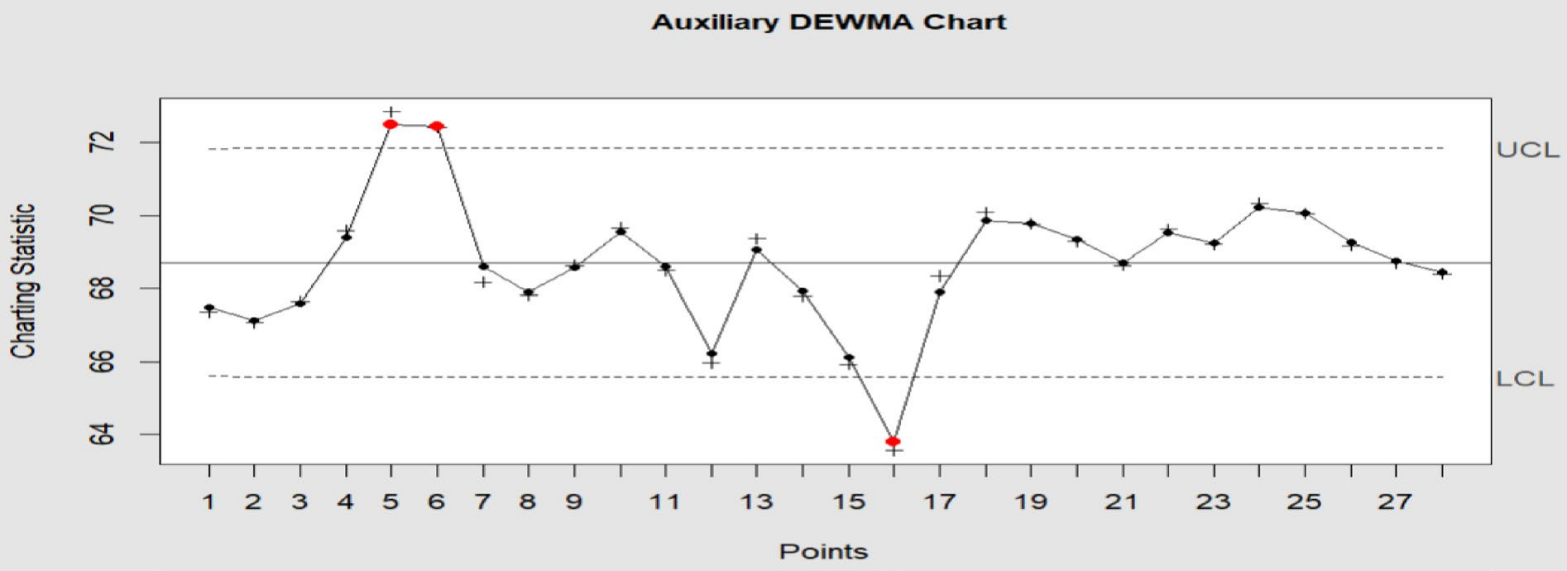
M_R_DEWMA control chart with time varying limits, parameters of chart $\rho_{VU} = 0.5$_,_ $\upsilon = 0.9$ and $L = 2.806$ at $ARL_{0} = 200$.

The study and auxiliary variable have a 0.5 correlation (i.e., $\rho_{VU} = 0.50$). In the comparison, we use the DEWMA and M_R_DEWMA charts. For the DEWMA chart with time-varying limits, we have utilized $\upsilon = 0.90$ and $L = 2.806$; similarly, for the M_R_DEWMA with time-varying limits, we have utilized $\upsilon = 0.90$ and $L = 2.806$ to produce two charts and an $ARL_{0} = 200$. The computations for the M_R_DEWMA chart, while Figs. 12, 13 provide the graphical representation of the two control charts. Plotting statistic for the DEWMA and M_R_DEWMA chart are $W_{t}$ and $C_{t}$ respectively. The proposed M_R_DEWMA chart clearly displays out-of-control signals at samples # 5, 6 and 16, as seen in Fig. 13. Three out-of-control signals in total can also be verified. The DEWMA control chart gives just one out-of-control signals at sample # 16. This shows how the proposed chart outperforms the DEWMA control chart. The proposed M_R_DEWMA control chart for the tensile dataset proves to be effective in identifying minor shifts in tensile strength values. This capability is vital in industrial settings where maintaining material quality within precise parameters is essential. By recognizing deviations promptly, manufacturers can implement corrective measures before the product’s strength drops below acceptable thresholds, thereby minimizing the chances of defects, recalls, or structural failures. From an operational perspective, this leads to cost savings, greater customer satisfaction, and increased reliability in processes.

## Conclusion

In this study, we propose a further advancement: the Modified Regression-Based DEWMA (M_R_DEWMA) control chart. This chart incorporates a regression estimator utilizing a single auxiliary variable to enhance monitoring efficiency. The performance of the proposed M_R_DEWMA chart is evaluated under both asymptotic and time-varying control limits. When the correlation between the study variable and the auxiliary variable is zero, the M_R_DEWMA and DEWMA charts become equivalent. The chart’s efficiency was assessed using key performance metrics, including ARL, SDRL, and MRL, across varying levels of correlation. A comparative analysis was conducted between the standard DEWMA chart, the M_R_DEWMA chart with asymptotic limits, and the M_R_DEWMA chart with time-varying limits. The findings demonstrate that the proposed M_R_DEWMA chart outperforms existing methods in detecting small to moderate shifts in the process mean, while maintaining satisfactory performance for larger shifts.

The improved M_R_DEWMA chart is particularly beneficial in industrial settings where early detection of small process shifts is critical. Practitioners are encouraged to adopt this chart for enhanced monitoring precision, reduced false alarms, and improved decision-making in quality control applications.

## Future recommendations

Future research may focus on the following directions to enhance and broaden the applicability of the proposed methodology:

- **Integration of Machine Learning Techniques:** Future studies can explore the incorporation of supervised and unsupervised machine learning algorithms (e.g., random forests, neural networks, and clustering methods) to better model the relationship between the process variable and auxiliary information. This integration may enhance the chart’s predictive capability and improve detection performance in complex industrial environments where patterns are nonlinear or data-driven insights are required.
- **Handling Non-Normal Distributions:** Real-world processes often deviate from the assumption of normality. Therefore, it is essential to investigate the performance and robustness of the M_R_DEWMA chart under non-normal conditions, such as skewed, heavy-tailed, or multimodal distributions. Future work may involve developing generalized charting schemes or applying transformation and bootstrapping techniques to maintain control performance in such settings.
- **Multivariate Extensions:** An important area for future exploration is the extension of the proposed methodology to multivariate process monitoring. This would involve the simultaneous evaluation of multiple interrelated quality characteristics using multivariate auxiliary information. Potential developments include the formulation of a multivariate M_R_DEWMA-type chart and assessment of its performance using metrics like multivariate ARL and T^2^ statistics.
- **Adaptive Control Limits:** Static control limits may not perform optimally in dynamic environments. Future research could focus on the development of adaptive control limit strategies that evolve based on real-time process behaviour and auxiliary data. Such adaptive mechanisms can improve responsiveness to shifts, reduce Type I error rates, and maintain long-term stability under varying process conditions.

## Supplementary Information

Supplementary Information.

## Data Availability

The data used to support the findings of this study are included within the article.
