# Supplementary material for: Enhanced DEWMA-type control chart for process mean monitoring utilizing auxiliary information
Source: Sci Rep. 2025 Dec 15;15:43765. doi: 10.1038/s41598-025-27540-6 (PMC12705690; doi:10.1038/s41598-025-27540-6)
Supplement: Supplementary file 1 — Supplementary Information. [file 41598_2025_27540_MOESM1_ESM.docx]

**Appendix A:**

Table 9: ARL, SDRL and MRL values for the M_R_ DEWMA Control Charts with time-varying control limits 𝛒_UV_=0.05

| **L** | **1.71** |  |  | **1.992** |  |  | **2.392** |  |  | **2.678** |  |  | **2.789** |  |  | **2.806** |  |  |
| --- | --- | --- | --- | --- | --- | --- | --- | --- | --- | --- | --- | --- | --- | --- | --- | --- | --- | --- |
| $\upsilon$ | **0.05** |  |  | **0.1** |  |  | **0.25** |  |  | **0.5** |  |  | **0.75** |  |  | **0.9** |  |  |
| **Shift** | **ARL** | **SDRL** | **MRL** | **ARL** | **SDRL** | **MRL** | **ARL** | **SDRL** | **MRL** | **ARL** | **SDRL** | **MRL** | **ARL** | **SDRL** | **MRL** | **ARL** | **SDRL** | **MRL** |
| **0** | 200.56 | 247.99 | 114 | 199.81 | 219.26 | 132 | 200.05 | 200.72 | 139 | 200.63 | 200.52 | 140 | 199.81 | 201.89 | 139 | 200.01 | 201.68 | 136 |
| **0.25** | 40.753 | 41.552 | 29 | 48.828 | 46.769 | 36 | 65.229 | 63.888 | 46 | 93.911 | 92.723 | 65 | 121.47 | 123.14 | 83 | 140.6 | 137.51 | 98 |
| **0.5** | 14.595 | 13.453 | 11 | 16.96 | 13.909 | 14 | 21.822 | 19.454 | 17 | 32.988 | 31.562 | 23 | 53.342 | 53.179 | 37 | 70.603 | 71.263 | 49 |
| **0.75** | 7.656 | 6.6633 | 6 | 8.8236 | 6.9651 | 7 | 10.390 | 7.8434 | 9 | 14.980 | 13.037 | 11 | 24.143 | 22.904 | 17 | 36.354 | 36.298 | 25 |
| **1** | 4.6374 | 3.9720 | 3 | 5.5404 | 4.1645 | 5 | 6.5302 | 4.5825 | 6 | 8.4186 | 6.6083 | 7 | 12.500 | 11.264 | 9 | 19.365 | 18.036 | 14 |
| **1.25** | 3.266 | 2.5927 | 2 | 3.8228 | 2.7997 | 3 | 4.6016 | 2.9698 | 4 | 5.4718 | 3.8181 | 4 | 7.6796 | 6.4394 | 6 | 11.030 | 10.420 | 8 |
| **1.5** | 2.4536 | 1.8002 | 2 | 2.8656 | 2.0171 | 2 | 3.4356 | 2.1408 | 3 | 3.9406 | 2.5472 | 3 | 5.1202 | 3.9075 | 4 | 7.2016 | 6.3694 | 5 |
| **1.75** | 1.9428 | 1.3070 | 1 | 2.2484 | 1.5113 | 2 | 2.6818 | 1.5952 | 2 | 3.0394 | 1.7348 | 3 | 3.656 | 2.5036 | 3 | 4.8688 | 3.9751 | 4 |
| **2** | 1.6306 | 0.9893 | 1 | 1.8554 | 1.2122 | 1 | 2.2086 | 1.5952 | 2 | 2.4748 | 1.3402 | 2 | 2.853 | 1.8123 | 2 | 3.5462 | 2.6646 | 3 |
| **2.25** | 1.423 | 0.7659 | 1 | 1.5854 | 0.8654 | 1 | 1.8604 | 0.9897 | 2 | 2.072 | 1.0633 | 2 | 2.3146 | 1.3087 | 2 | 2.7636 | 1.9385 | 2 |
| **2.5** | 1.2624 | 0.5734 | 1 | 1.3772 | 0.6769 | 1 | 1.6068 | 0.7879 | 1 | 1.8082 | 0.8702 | 2 | 1.9426 | 1.0256 | 2 | 2.1676 | 1.3899 | 2 |
| **2.75** | 1.168 | 0.4467 | 1 | 1.2586 | 0.5297 | 1 | 1.4262 | 0.668 | 1 | 1.5882 | 0.7185 | 1 | 1.685 | 0.8376 | 2 | 1.8244 | 1.0975 | 2 |
| **3** | 1.1078 | 0.3449 | 1 | 1.692 | 0.4183 | 1 | 1.2978 | 0.5437 | 1 | 1.4244 | 0.6063 | 1 | 1.495 | 0.6800 | 1 | 1.574 | 0.8604 | 1 |

Table 10: ARL, SDRL and MRL values for the M_R_ DEWMA Control Charts with time-varying control limits 𝛒_UV_=0.90

| **L** | **1.71** |  |  | **1.992** |  |  | **2.392** |  |  | **2.678** |  |  | **2.789** |  |  | **2.806** |  |  |
| --- | --- | --- | --- | --- | --- | --- | --- | --- | --- | --- | --- | --- | --- | --- | --- | --- | --- | --- |
| $\upsilon$ | **0.05** |  |  | **0.1** |  |  | **0.25** |  |  | **0.5** |  |  | **0.75** |  |  | **0.9** |  |  |
| **Shift** | **ARL** | **SDRL** | **MRL** | **ARL** | **SDRL** | **MRL** | **ARL** | **SDRL** | **MRL** | **ARL** | **SDRL** | **MRL** | **ARL** | **SDRL** | **MRL** | **ARL** | **SDRL** | **MRL** |
| **0** | 200.7 | 248.38 | 114 | 199.53 | 215.71 | 131 | 200.79 | 204.45 | 141 | 199.834 | 203.185 | 136 | 201.05 | 201.17 | 141 | 199.81 | 193.95 | 140 |
| **0.25** | 11.851 | 10.746 | 9 | 13.953 | 11.389 | 11 | 17.215 | 14.165 | 13 | 26.018 | 23.642 | 19 | 41.193 | 40.412 | 29 | 57.644 | 56.957 | 40 |
| **0.5** | 3.772 | 3.144 | 3 | 4.517 | 3.405 | 4 | 5.390 | 3.551 | 5 | 6.523 | 4.812 | 5 | 9.601 | 8.131 | 7 | 14.143 | 13.152 | 10 |
| **0.75** | 2.0146 | 1.3756 | 2 | 2.3356 | 1.5734 | 2 | 2.781 | 1.6895 | 2 | 3.1506 | 1.8302 | 3 | 3.8662 | 2.7497 | 3 | 5.189 | 4.368 | 4 |
| **1** | 1.3862 | 0.734 | 1 | 1.5712 | 0.8694 | 1 | 1.809 | 0.945 | 2 | 2.0532 | 1.0343 | 2 | 2.2458 | 1.2785 | 2 | 2.665 | 1.9132 | 2 |
| **1.25** | 1.139 | 0.3976 | 1 | 1.2164 | 0.496 | 1 | 1.374 | 0.61204 | 1 | 1.5104 | 0.6642 | 1 | 1.6034 | 0.7498 | 1 | 1.7336 | 0.9808 | 1 |
| **1.5** | 1.0414 | 0.2061 | 1 | 1.0762 | 0.2779 | 1 | 1.1608 | 0.39112 | 1 | 1.2346 | 0.4586 | 1 | 1.283 | 0.5037 | 1 | 1.312 | 0.5758 | 1 |
| **1.75** | 1.0098 | 0.09852 | 1 | 1.022 | 0.1507 | 1 | 1.0552 | 0.23187 | 1 | 1.0876 | 0.2869 | 1 | 1.1118 | 0.3288 | 1 | 1.1152 | 0.3376 | 1 |
| **2** | 1.0018 | 0.04239 | 1 | 1.004 | 0.0631 | 1 | 1.014 | 0.1175 | 1 | 1.032 | 0.17715 | 1 | 1.036 | 0.1873 | 1 | 1.0416 | 0.2007 | 1 |
| **2.25** | 1.0002 | 0.01414 | 1 | 1.0008 | 0.0283 | 1 | 1.003 | 0.05469 | 1 | 1.0052 | 0.07193 | 1 | 1.0104 | 0.10146 | 1 | 1.01 | 0.09951 | 1 |
| **2.5** | 1 | 0.0003 | 1 | 1 | 0.00032 | 1 | 1.0004 | 0.01999 | 1 | 1.0016 | 0.03997 | 1 | 1.002 | 0.0447 | 1 | 1.0008 | 0.0283 | 1 |
| **2.75** | 1 | 0.000013 | 1 | 1 | 0.00004 | 1 | 1 | 0.00103 | 1 | 1.0004 | 0.01999 | 1 | 1.0002 | 0.01414 | 1 | 1.0006 | 0.0245 | 1 |
| **3** | 1 | 0.000006 | 1 | 1 | 0.000003 | 1 | 1 | 0.0001 | 1 | 1 | 0.00008 | 1 | 1 | 0.00003 | 1 | 1 | 0.00007 | 1 |

Table 11: ARL, SDRL and MRL values for the M_R_ DEWMA Control Charts with asymptotic control limits 𝛒_UV_=0.05

| **L** | **1.71** |  |  | **1.992** |  |  | **2.392** |  |  | **2.678** |  |  | **2.789** |  |  | **2.806** |  |  |
| --- | --- | --- | --- | --- | --- | --- | --- | --- | --- | --- | --- | --- | --- | --- | --- | --- | --- | --- |
| $\upsilon$ | **0.05** |  |  | **0.1** |  |  | **0.25** |  |  | **0.5** |  |  | **0.75** |  |  | **0.9** |  |  |
| **Shift** | **ARL** | **SDRL** | **MRL** | **ARL** | **SDRL** | **MRL** | **ARL** | **SDRL** | **MRL** | **ARL** | **SDRL** | **MRL** | **ARL** | **SDRL** | **MRL** | **ARL** | **SDRL** | **MRL** |
| **0** | 200.9 | 183.49 | 142 | 200.5 | 185.46 | 146 | 200.21 | 196.55 | 138 | 200.28 | 195.87 | 141 | 200.80 | 197.98 | 142 | 200.87 | 200.08 | 138 |
| **0.25** | 53.741 | 34.598 | 44 | 55.17 | 42.337 | 43 | 70.016 | 63.614 | 51 | 96.301 | 91.959 | 69 | 125.98 | 121.18 | 91 | 146.16 | 145.07 | 102 |
| **0.5** | 25.946 | 9.680 | 24 | 22.54 | 11.687 | 19 | 23.795 | 18.273 | 18 | 34.023 | 30.307 | 25 | 54.268 | 53.019 | 38 | 72.361 | 71.171 | 51 |
| **0.75** | 18.501 | 4.693 | 18 | 14.56 | 5.346 | 13 | 12.553 | 7.504 | 10.5 | 15.446 | 12.739 | 11 | 24.653 | 23.274 | 18 | 36.918 | 35.608 | 26 |
| **1** | 14.999 | 2.9918 | 15 | 11.21 | 3.007 | 11 | 8.4406 | 3.857 | 7 | 8.911 | 6.3712 | 7 | 13.211 | 11.408 | 10 | 20.091 | 19.679 | 14 |
| **1.25** | 12.947 | 2.0762 | 13 | 9.391 | 2.0313 | 9 | 6.5096 | 2.4344 | 6 | 6.011 | 3.668 | 5 | 7.916 | 6.5246 | 6 | 11.440 | 10.503 | 8 |
| **1.5** | 11.488 | 1.5798 | 11 | 8.218 | 1.5162 | 8 | 5.3942 | 1.6292 | 5 | 4.4882 | 2.2041 | 4 | 5.394 | 4.0003 | 4 | 7.243 | 6.2276 | 5 |
| **1.75** | 10.423 | 1.3007 | 10 | 7.371 | 1.1828 | 7 | 4.6748 | 1.2031 | 4 | 3.6114 | 1.5644 | 3 | 3.9118 | 2.5592 | 3 | 4.9666 | 4.1305 | 4 |
| **2** | 9.6158 | 1.0722 | 10 | 6.758 | 0.9933 | 7 | 4.1644 | 0.9453 | 4 | 3.0878 | 1.1608 | 3 | 3.0984 | 1.8095 | 3 | 3.5986 | 2.7603 | 3 |
| **2.25** | 8.9762 | 0.9240 | 9 | 6.242 | 0.8228 | 6 | 3.7866 | 0.7777 | 4 | 2.7178 | 0.8799 | 3 | 2.4922 | 1.2978 | 2 | 2.7796 | 1.9126 | 2 |
| **2.5** | 8.4138 | 0.8084 | 8 | 5.831 | 0.7417 | 6 | 3.5086 | 0.6545 | 3 | 2.4434 | 0.6932 | 2 | 2.149 | 1.0009 | 2 | 2.243 | 1.4417 | 2 |
| **2.75** | 7.9648 | 0.7328 | 8 | 5.487 | 0.6624 | 5 | 3.2808 | 0.5593 | 3 | 2.2482 | 0.5785 | 2 | 1.8744 | 0.8229 | 2 | 1.876 | 1.1335 | 2 |
| **3** | 7.5758 | 0.6557 | 8 | 5.222 | 0.5792 | 5 | 3.1006 | 0.4981 | 3 | 2.1082 | 0.4755 | 2 | 1.6756 | 0.6926 | 2 | 1.6166 | 0.8543 | 1 |

| **L** | **1.71** |  |  | **1.992** |  |  | **2.392** |  |  | **2.678** |  |  | **2.789** |  |  | **2.806** |  |  |
| --- | --- | --- | --- | --- | --- | --- | --- | --- | --- | --- | --- | --- | --- | --- | --- | --- | --- | --- |
| $\upsilon$ | **0.05** |  |  | **0.1** |  |  | **0.25** |  |  | **0.5** |  |  | **0.75** |  |  | **0.9** |  |  |
| **Shift** | **ARL** | **SDRL** | **MRL** | **ARL** | **SDRL** | **MRL** | **ARL** | **SDRL** | **MRL** | **ARL** | **SDRL** | **MRL** | **ARL** | **SDRL** | **MRL** | **ARL** | **SDRL** | **MRL** |
| **0** | 200.88 | 180.5 | 145 | 200.34 | 186.84 | 145 | 200.06 | 196.24 | 139.5 | 200.59 | 198.44 | 138 | 200.19 | 201.36 | 137 | 200.92 | 198.51 | 140 |
| **0.25** | 23.370 | 7.769 | 22 | 19.515 | 9.0431 | 17 | 19.586 | 14.252 | 15 | 26.985 | 23.801 | 20 | 42.281 | 40.756 | 30 | 59.804 | 59.737 | 41 |
| **0.5** | 13.777 | 2.464 | 13 | 10.059 | 2.3302 | 10 | 7.1688 | 2.9194 | 6 | 7.109 | 4.7035 | 6 | 9.7526 | 8.2638 | 7 | 14.6138 | 13.8125 | 10 |
| **0.75** | 10.610 | 1.362 | 10 | 7.4962 | 1.216 | 7 | 4.742 | 1.2197 | 5 | 3.75 | 1.656 | 3 | 4.0902 | 2.7506 | 3 | 5.3062 | 4.34581 | 4 |
| **1** | 8.8994 | 0.914 | 9 | 6.1846 | 0.8257 | 6 | 3.7472 | 0.7641 | 4 | 2.6688 | 0.8417 | 3 | 2.441 | 1.2481 | 2 | 2.6964 | 1.8351 | 2 |
| **1.25** | 7.802 | 0.699 | 8 | 5.3854 | 0.6332 | 5 | 3.1894 | 0.5157 | 3 | 2.1994 | 0.5416 | 2 | 1.7934 | 0.7789 | 2 | 1.7644 | 0.9929 | 2 |
| **1.5** | 7.0262 | 0.575 | 7 | 4.8246 | 0.5249 | 5 | 2.8712 | 0.5045 | 3 | 1.928 | 0.5016 | 2 | 1.426 | 0.5605 | 1 | 1.3362 | 0.5798 | 1 |
| **1.75** | 6.412 | 0.519 | 6 | 4.3582 | 0.4861 | 4 | 2.5484 | 0.4541 | 3 | 1.7298 | 0.4605 | 2 | 1.2046 | 0.4147 | 1 | 1.1308 | 0.3568 | 1 |
| **2** | 6.0004 | 0.492 | 6 | 4.0574 | 0.4842 | 4 | 2.2408 | 0.4281 | 2 | 1.5006 | 0.4516 | 1 | 1.0842 | 0.2778 | 1 | 1.0482 | 0.2151 | 1 |
| **2.25** | 5.6098 | 0.376 | 6 | 3.902 | 0.4487 | 4 | 2.0666 | 0.2492 | 2 | 1.2854 | 0.4055 | 1 | 1.0224 | 0.1479 | 1 | 1.0186 | 0.0923 | 1 |
| **2.5** | 5.1842 | 0.388 | 5 | 3.6266 | 0.3188 | 4 | 2.0111 | 0.1043 | 2 | 1.1194 | 0.3243 | 1 | 1.005 | 0.0705 | 1 | 1.0096 | 0.0509 | 1 |
| **2.75** | 5.014 | 0.146 | 5 | 3.2794 | 0.2887 | 3 | 2.0006 | 0.0245 | 2 | 1.0436 | 0.2042 | 1 | 1.001 | 0.0316 | 1 | 1.0008 | 0.01414 | 1 |
| **3** | 4.9338 | 0.252 | 5 | 3.0586 | 0.2349 | 3 | 1.9998 | 0.0141 | 2 | 1.0098 | 0.0985 | 1 | 1 | 0.0026 | 1 | 1 | 0.00211 | 1 |

Table 12: ARL, SDRL and MRL values for the M_R_ DEWMA Control Charts with asymptotic control limits 𝛒_UV_=0.90

**Appendix B:**

**Mean and Variance of Regression Estimator:**

$$M_{R}=\overline{U}+b_{UV}(\mu_{V}-\overline{V})$$

$$M_{R}=\overline{U}(1+\mathrm{e}_{U})+\mathrm{b}_{UV}(\overline{V}-\overline{V}(1+\mathrm{e}_{V}))$$

$$=\overline{U}+\overline{U}e_{U}+b_{UV}(\overline{V}-\overline{V}-\overline{V}\mathrm{e}_{V})$$

$$=\overline{U}+\overline{U}e_{U}-\overline{V}b_{UV}e_{V}$$

$$M_{R}-\overline{U}=\overline{U}e_{U}-\overline{V}b_{UV}e_{V}$$

$$E(M_{R}-\overline{U})=\mathrm{E}(\overline{U}e_{U}-\overline{V}b_{UV}e_{V})$$

$$E(\mathrm{M}_{R})=\overline{U}$$

For variance, we proceed as

$$(M_{R}-\overline{U})^{2}=(\overline{U}e_{U}-\overline{V}b_{UV}e_{V})^{2}$$

$$=\overline{U}^{2}e_{U}^{2}+b_{UV}^{2}\overline{V}^{2}e_{V}^{2}-2\overline{U}\overline{V}b_{UV}e_{V}e_{U}$$

$$E(M_{R}-\overline{U})^{2}=E(\overline{U}^{2}e_{U}^{2}+b_{UV}^{2}\overline{V}^{2}e_{V}^{2}-2\overline{U}\overline{V}b_{UV}e_{V}e_{U})$$

$$Var(\mathrm{M}_{R})=\overline{U}^{2}C_{U}^{2}+\overline{V}^{2}b_{UV}^{2}C_{V}^{2}-2\overline{U}\overline{V}b_{UV}C_{UV}$$

$$\therefore C_{U}^{2}=\frac{\sigma_{U}^{2}}{\overline{U}};C_{V}^{2}=\frac{\sigma_{V}^{2}}{\overline{V}};C_{UV}=\frac{\sigma_{UV}}{\overline{U}\overline{V}}$$

$$=\sigma_{U}^{2}+b_{UV}^{2}\sigma_{V}^{2}-2\sigma_{UV}b_{UV}$$

$$\therefore b_{UV}=\rho_{UV}\left(\frac{\sigma_{U}}{\sigma_{V}}\right)$$

$$=\sigma_{U}^{2}+\rho_{UV}^{2}\sigma_{U}^{2}-2\rho_{UV}^{2}\sigma_{U}^{2}$$

$$\mathrm{Var}(\mathrm{M}_{R})=\sigma_{U}^{2}(1-\rho_{UV}^{2})$$

**Properties of the proposed M_R_DEWMA Chart:**

$$C_{t}=\upsilon H_{t}+(1-\upsilon)\mathrm{C}_{(\mathrm{t}-1)}$$

$$=\upsilon H_{t}+(1-\upsilon)[\upsilon C_{(\mathrm{t}-1)}+(1-\upsilon)\mathrm{C}_{(\mathrm{t}-2)}]$$

$$=\upsilon\sum_{i=1}^{t} (1-\upsilon)^{(\mathrm{t}-1)}H_{t}+(1-\upsilon)^{t}C_{0}$$

$$=\upsilon\sum_{i=1}^{t} (1-\upsilon)^{(\mathrm{t}-1)}[\upsilon\sum_{j=1}^{i} (1-\upsilon)^{(\mathrm{i}-\mathrm{j})}M_{R_{j}}+(1-\upsilon)^{i}H_{0}]+(1-\upsilon)^{t}C_{0}$$

$$=\upsilon^{2}\sum_{i=1}^{t} \sum_{j=1}^{i} (1-\upsilon)^{t-j}M_{R_{j}}+\upsilon(1-\upsilon)^{i}H_{0}+(1-\upsilon)^{t}C_{0}$$

$$\therefore C_{0}=H_{0}=\mu_{0}$$

$$=\upsilon^{2}\sum_{j=1}^{t} (\mathrm{t}-\mathrm{j}+1)(1-\upsilon)^{t-j}M_{R_{j}}+(1-\upsilon)^{t}(\upsilon\mathrm{t}+1)\mu_{0}$$

$$E(\mathrm{C}_{t})=E[\upsilon^{2}\sum_{j=1}^{t} (\mathrm{t}-\mathrm{j}+1)(1-\upsilon)^{t-j}M_{R_{j}}+(1-\upsilon)^{t}(\upsilon\mathrm{t}+1)\mu_{0}]$$

$$\therefore E(\mathrm{M}_{R_{j}})=\mathrm{M}_{R_{0}}=\mu_{0}$$

$$=[\upsilon^{2}\sum_{j=1}^{t} (\mathrm{t}-\mathrm{j}+1)(1-\upsilon)^{t-j}+(1-\upsilon)^{t}(\upsilon\mathrm{t}+1)]\mu_{0}$$

$$=\left[\frac{\upsilon^{2}}{(1-\upsilon)}\sum_{j=1}^{t} (\mathrm{t}-\mathrm{j}+1)(1-\upsilon)^{t-j+1}+(1-\upsilon)^{t}(\upsilon\mathrm{t}+1)\right]\mu_{0}$$

$$=\left[\frac{\upsilon^{2}}{(1-\upsilon)}\sum_{j=1}^{t} P(1-\upsilon)^{P}+(1-\upsilon)^{t}(\upsilon\mathrm{t}+1)\right]\mu_{0}$$

$$\therefore\sum_{k=1}^{n} ka^{k}=\frac{a(1-\mathrm{a}^{n})}{(1-\mathrm{a})^{2}}-\frac{na^{n+1}}{(1-\mathrm{a})}$$

$$=\left[\frac{\upsilon^{2}}{(1-\upsilon)}\left(\frac{(1-\upsilon)(1-(1-\upsilon)^{t})}{(1-(1-\upsilon))^{2}}-\frac{t(1-\upsilon)^{t+1}}{(1-(1-\upsilon))}\right)+(1-\upsilon)^{t}(\upsilon\mathrm{t}+1)\right]\mu_{0}$$

$$=\left[\upsilon^{2}\left(\frac{(1-(1-\upsilon)^{t})}{\upsilon^{2}}-\frac{t(1-\upsilon)^{t}}{\upsilon}\right)+(1-\upsilon)^{t}(\upsilon\mathrm{t}+1)\right]\mu_{0}$$

$$=\left[1-(1-\upsilon)^{t}-\upsilon t(1-\upsilon)^{t}+(1-\upsilon)^{t}(\upsilon\mathrm{t}+1)\right]\mu_{0}$$

$$E(\mathrm{C}_{t})=\mu_{0}$$

For variance, we proceed as

$$\mathrm{Var}(\mathrm{C}_{t})=Var\left[\upsilon^{2}\sum_{j=1}^{t} (\mathrm{t}-\mathrm{j}+1)(1-\upsilon)^{t-j}M_{R_{j}}+(1-\upsilon)^{t}(\upsilon\mathrm{t}+1)\mu_{0}\right]$$

$$=\upsilon^{4}\sum_{j=1}^{t} (\mathrm{t}-\mathrm{j}+1)^{2}(1-\upsilon)^{2(t-j)}\mathrm{Var}(M_{R_{j}})+(1-\upsilon)^{2t}(\upsilon\mathrm{t}+1)^{2}\mathrm{Var}(\mu_{0})$$

$$\therefore Var\left(M_{R_{j}}\right)=\sigma_{0}^{2};\mathrm{Var}(\mu_{0})=0$$

$$=\upsilon^{4}\sum_{j=1}^{t} (\mathrm{t}-\mathrm{j}+1)^{2}(1-\upsilon)^{2(t-j)}\sigma_{0}^{2}$$

$$=\frac{\upsilon^{4}}{(1-\upsilon)^{2}}\sum_{j=1}^{t} (\mathrm{t}-\mathrm{j}+1)^{2}(1-\upsilon)^{2(t-j+1)}\sigma_{0}^{2}$$

$$=\frac{\upsilon^{4}}{(1-\upsilon)^{2}}\sum_{j=1}^{t} (\mathrm{u})^{2}(1-\upsilon)^{2\mathrm{u}}\sigma_{0}^{2}$$

$$\therefore\sum_{k=1}^{n} k^{2}a^{k}=\frac{a+a^{2}-(\mathrm{n}+1)^{2}a^{n+1}+(2\mathrm{n}^{2}+2\mathrm{n}-1)\mathrm{a}^{n+2}-n^{2}a^{n+3}}{(1-\mathrm{a})^{3}}$$

$$=\frac{\upsilon^{4}}{(1-\upsilon)^{2}}\left[\frac{1}{(1-(1-\upsilon)^{2})^{3}}\left((1-\upsilon)^{2}+(1-\upsilon)^{4}-(\mathrm{t}+1)^{2}(1-\upsilon)^{2(\mathrm{t}+1)}+(2t^{2}+2\mathrm{t}-1)(1-\upsilon)^{2(\mathrm{t}+2)}-t^{2}(1-\upsilon)^{2(\mathrm{t}+3)}\right)\right]$$

$$\mathrm{Var}(\mathrm{C}_{t})=\frac{\upsilon^{4}}{(1-(1-\upsilon)^{2})^{3}}\left[1+(1-\upsilon)^{2}-(\mathrm{t}+1)^{2}(1-\upsilon)^{2t}+(2\mathrm{t}^{2}+2\mathrm{t}-1)(1-\upsilon)^{2(\mathrm{t}+1)}-t^{2}(1-\upsilon)^{2t+4}\right]$$

If $t\to\infty$the variance will becomes:

$$\mathrm{Var}(\mathrm{C}_{t})=\frac{\upsilon(2-2\upsilon+\upsilon^{2})}{(2-\upsilon)^{3}}$$
